# Evaluation and Application of Population Pharmacokinetic Models for Identifying Delayed Methotrexate Elimination in Patients With Primary Central Nervous System Lymphoma

**DOI:** 10.3389/fphar.2022.817673

**Published:** 2022-03-09

**Authors:** Junjun Mao, Qing Li, Pei Li, Weiwei Qin, Bobin Chen, Mingkang Zhong

**Affiliations:** ^1^ Department of Pharmacy, Huashan Hospital, Fudan University, Shanghai, China; ^2^ Department of Hematology, Huashan Hospital, Fudan University, Shanghai, China; ^3^ Department of Hematology, Huashan Hospital North, Fudan University, Shanghai, China

**Keywords:** methotrexate, population pharmacokinetics, external evaluation, delayed elimination, Monte carlo simulations

## Abstract

**Objective:** Several population pharmacokinetic (popPK) models have been developed to determine the sources of methotrexate (MTX) PK variability. It remains unknown if these published models are precise enough for use or if a new model needs to be built. The aims of this study were to 1) assess the predictability of published models and 2) analyze the potential risk factors for delayed MTX elimination.

**Methods:** A total of 1458 MTX plasma concentrations, including 377 courses (1–17 per patient), were collected from 77 patients who were receiving high-dose MTX for the treatment of primary central nervous system lymphoma in Huashan Hospital. PopPK analysis was performed using the NONMEM® software package. Previously published popPK models were selected and rebuilt. A new popPK model was then constructed to screen potential covariates using a stepwise approach. The covariates were included based on the combination of theoretical mechanisms and data properties. Goodness-of-fit plots, bootstrap, and prediction- and simulation-based diagnostics were used to determine the stability and predictive performance of both the published and newly built models. Monte Carlo simulations were conducted to qualify the influence of risk factors on the incidence of delayed elimination.

**Results:** Among the eight evaluated published models, none presented acceptable values of bias or inaccuracy. A two-compartment model was employed in the newly built model to describe the PK of MTX. The estimated mean clearance (CL/*F*) was 4.91 L h^−1^ (relative standard error: 3.7%). Creatinine clearance, albumin, and age were identified as covariates of MTX CL/*F*. The median and median absolute prediction errors of the final model were -10.2 and 36.4%, respectively. Results of goodness-of-fit plots, bootstrap, and prediction-corrected visual predictive checks indicated the high predictability of the final model.

**Conclusions:** Current published models are not sufficiently reliable for cross-center use. The elderly patients and those with renal dysfunction, hypoalbuminemia are at higher risk of delayed elimination.

## 1 Introduction

High-dose methotrexate (HD-MTX, ≥ 1 g m^−2^) is the base therapy for the treatment of various lymphoid malignancies, such as acute lymphoblastic leukemia (Sakura et al., 2018) and non-Hodgkin’s lymphoma (Reiter et al., 1999), especially for treating primary or secondary central nervous system lymphoma (Plotkin et al., 2004; Zhu et al., 2009). As an antifolate inhibitor of dihydrofolate reductase, MTX may cause the depletion of purines and thymidylate, which inhibits DNA synthesis, leading to cell death (Baram et al., 1987).

Following intravenous infusion, approximately 60% of MTX binds to plasma protein (Maia et al., 1996). As a small polar molecule, MTX elimination is highly correlated with renal function. Approximately only 10% of MTX is excreted as unchanged drug in the bile, whereas the majority is eliminated as unchanged drug through the kidneys within 24 h (Csordas et al., 2013).

Both the pharmacokinetic (PK) of MTX, which exhibit wide inter-individual variability (IIV), and its exposure are directly related to efficacy and toxicity (Evans et al., 1986; Evans et al., 1998). Patients experiencing delayed MTX elimination have been reported to be at an elevated risk of toxicity, such as nephrotoxicity, myelotoxicity, mucositis, neurological complications, and other adverse effects, which may lead to significant morbidity and delays in treatment (Howard et al., 2016). To prevent this systemic toxicity, supportive care, such as fluid hydration, urine alkalinization, and leucovorin rescue, is conducted as HD-MTX is administered (Widemann and Adamson, 2006). Post-dose therapeutic drug monitoring is routinely performed to maintain MTX plasma concentrations within the cytotoxic range for leukemic cells and, below those associated with toxicity (Wall et al., 2000; Paci et al., 2014).

Delayed MTX elimination is defined as plasma MTX concentrations above 50 μmol L^−1^ at 24 h, above 5 μmol L^−1^ at 48 h, or above 0.2 μmol L^−1^ at 72 h (Hospira, 2017). As MTX-induced nephrotoxicity correlates with clearance (CL), delayed elimination is closely related to acute kidney injury (Huang et al., 2020). Thus, if patients with delayed MTX CL can be identified, allowing for the implementation of a personalized dosage regimen before chemotherapy, concentration-related toxicity may be avoided (Ramsey et al., 2018). Although some oncology guidelines recommend MTX dosage based on the patient’s body size (i.e., body surface area or actual body weight) (Gurney and Shaw, 2007; Griggs et al., 2012), this strategy is not always suitable for clinical practice, particularly for obese patients (Gallais et al., 2020; Pai et al., 2020). Therefore, determining the risk factors for delayed MTX elimination is essential.

Compared to conventional PK analysis, population pharmacokinetics (popPK) is a superior approach in facilitating the understanding and quantification of PK variability (Ette and Williams, 2004). PopPK models combined with maximum posterior Bayesian estimation can be used to guide dosing regimen individualization.

A few popPK models have been constructed to assess the sources of MTX PK variability. However, whether these popPK models can be extrapolated to other clinical centers remains unknown. In addition, inconsistencies and differences in study design, research purpose, and population properties have been noted in some of the published popPK models with regard to model structure, parameter estimates, selected covariates, and their functional forms on PK parameters (Mao et al., 2018; Cheng et al., 2020). As the published models were developed based on specific populations, selecting an appropriate model to guide precision dosing in clinical practice is challenging.

Whether the published models are sufficiently precise for use in patients with primary central nervous system lymphoma, or whether a new popPK model based on our center is needed, remains unknown. Therefore, we summarized and assessed the predictability of published HD-MTX popPK models in adult patients with lymphoid malignancies. In addition, a new popPK model was constructed to investigate the effects of physic-pathological parameters on the distribution and elimination of MTX. The predictability of this model was also compared with previously published models. Finally, the most suitable model was applied to identify the population with delayed elimination.

## 2 Materials and Methods

### 2.1 Patient Data Collection

Data from 77 adults (49 men and 28 women) who were diagnosed with primary central nervous system lymphoma and received HD-MTX (>1 g m^−2^) for treatment between June 2011 and November 2016 were retrospectively collected.

To protect against MTX-induced renal dysfunction, hydration and alkalization (urine pH > 7) were achieved 12 h prior to initiating MTX therapy (Hospira, 2017). Serial plasma MTX levels were measured at 24, 48, and 72 h until the plasma concentration was ≤0.2 μmol L^−1^ (Dupuis et al., 2008). The leucovorin rescue was initiated and repeated every 6 h after 24 h from the MTX infusion until the MTX concentration was lower than ≤0.2 μmol L^−1^.

MTX concentrations in plasma were determined using a well-validated enzyme-multiplied immunoassay (EMIT) using the SYVA Viva-Emit 2000 Kit (Siemens Healthcare Diagnostics, Newark, DE, United States). The limit of detection was 0.3 μmol L^−1^, and the calibration concentrations ranged from 0.3 μmol L^−1^–2,600 μmol L^−1^.

Demographic covariates, including age, sex, weight, body surface area (BSA), and concomitant medication with benzimidazoles and corticoids, were included in the database. Clinical covariates, such as serum creatinine (SCr) levels, were recorded before each MTX infusion and in tandem with MTX plasma samples. According to the standard of ‘drug interaction score’ (Benz-de Bretagne et al., 2014), benzimidazoles and corticoids were assigned scores of 2 and 1, respectively.

The Ethics Committee of Huashan Hospital approved the study protocols. Written informed consent was obtained from all volunteers.

### 2.2 Population Pharmacokinetic Models

Our study consisted of the following steps:

Step 1: PopPK models of HD-MTX in adult patients with lymphoid malignancies were selected and reviewed.

Step 2: A new popPK model was constructed based on our dataset.

Step 3: The predictability of published popPK models and that of the newly built model was evaluated.

Step 4: The most suitable model was applied to identify the population with delayed elimination.

#### 2.2.1 Review of Published popPK Studies on HD-MTX

A systematic review of popPK studies on HD-MTX published in English before 31 December 2020 was performed using PubMed, Web of Science, and Embase. According to the Preferred Reporting Items for Systematic Reviews and Meta-analyses statement, the relevant identification, screening, and assessment were conducted (Moher et al., 2015).

The inclusion criteria for published studies were as follows: 1) studied population: adult patients with lymphoid malignancies; 2) treatment: HD-MTX; 3) PK analysis using NONMEM® software package; and 4) language: English. Studies were excluded if they 1) lacked the required details for external evaluation and re-estimate or 2) overlapped with other data or cohorts. Reference lists of the identified reports were also screened.

The demographic characteristics and following popPK parameters were collected from each identified study: apparent clearance (CL/*F*), apparent volume of distribution (V/*F*), and corresponding between-subject variability and residual variability.

#### 2.2.2 Development of a Model Based on Our Dataset

NONMEM® software package (version 7.4; ICON Development Solutions, Ellicott City, MD, United States) with Pirana® 2.9 as an interface for Perl Speaks NONMEM (PsN; version 4.9.0) was used for popPK analysis (Keizer et al., 2013). R software (version 3.5.0, http://www.r-project.org/) was used to construct the visualizations for output and model evaluations.

Based on a literature review and visual data inspection, the concentration-time profile of MTX was described by two-compartment models. Model variability and random effects were classified as one of three types of errors: between-subject variability (BSV), inter-occasion variability (IOV), and residual unexplained variability (RUV). BSV was assumed to be log-normally distributed and an estimate for all parameters. IOV was assumed to be the same for all occasions (Karlsson and Sheiner, 1993). RUV was described by testing proportional and combined proportional as well as additive structures. The first-order conditional estimation method including η-ε interaction (FOCE-I) was used for the model (Beal et al., 1989).

The evaluated covariates included demographic and pathophysiological data, as well as concomitant medications (Table 1). Age, body size, hematocrit (HCT), albumin (ALB), creatinine clearance (CrCL), and concomitant medications were evaluated as possible covariates of MTX PK. CrCL was estimated using the Cockcroft-Gault equation (Cockcroft and Gault, 1976). As the most frequently identified covariate, the effect of CrCL on MTX PK CL/*F* was tested first. The other covariates were screened according to a previous study and their clinical relevance (Mao et al., 2018). Each co-administered drug was assigned a “drug interaction score (DIS)” list (Supplementary Table S1), as presented previously (Benz-de Bretagne et al., 2014), and was considered individually by testing its effect on PK parameters as categorical variable.

**TABLE 1:** Patient characteristics used to develop and evaluate population model.

Characteristics	Number or mean ± SD	Median (range)
No. of patients (Male/Female)^a^	77 (49/28)	/
No. of Samples^b^	1,458	/
Age (years)	54.6 ± 9.2	56 (28–76)
Height (cm)	169 ± 7	170 (150–185)
Weight (kg)	67.8 ± 10.7	69.0 (41.0–94.0)
Body surface area (m^2^)	1.60 ± 0.30	1.61 (0.85–2.32)
Methotrexate dose (g)	4.7 ± 1.9	4.0 (2.0–15.8)
Methotrexate dose (g m^−2^)	3.0 ± 1.3	2.8 (1.1–10.2)
Dosing time (h)	3.9 ± 3.7	3 (1–28.25)
Occasions (n)	4.9 ± 3.6	4 (1–17)
Samples per individual (n)	19.0 ± 13.7	16 (3–67)
Hematocrit (%)	36.0 ± 4.5	36.1 (15.7–48.4)
Total Bilirubin (μmol L^−1^)	9.8 ± 3.9	9.5 (3.1–36.1)
Alanine aminotransferase (U L^−1^)	38.2 ± 39.5	28.0 (4.0–420.0)
Aspartate transferase (U L^−1^)	24.7 ± 22.0	20.0 (5.0–559.0)
Albumin (g L^−1^)	38.7 ± 4.3	39.0 (24.0–50.0)
Total protein (g L^−1^)	64.2 ± 6.3	64.0 (41.0–82.0)
Serum Creatinine (μmol L^−1^)	70.5 ± 28.7	66.0 (22.0–480.0)
Creatinine Clearance (ml min^−1^)^c^	104.2 ± 34.2	98 (15.1–326.5)
**Concomitant medications** ^b^		
Omeprazole	214	/
Esomeprazole	19	/
Lanzoprazole	969	/
Pantoprazole	58	/
Dexamethasone	1,122	/

^a^Data are expressed as number of patients.

^b^Data are xpressed as number of samples.

^c^Calculate following the Cockcroft-Gault formula: CrCL = [(140-Age (year)) ×WT (kg)]/(0.818×Scr (μmol L^−1^)) × (0.85 for female).

The influence of continuous covariates was explored using the linear, exponential, and power function models, whereas that of categorical variables was described using a shift model. After considering the most frequently identified covariates in the model, the remaining covariates were screened using a stepwise approach primarily based on objective function value (OFV) (Beal et al., 1989), and parameter precision. Error estimates were also considered.

The likelihood ratio tests at a significance level of *p* < 0.05 (ΔOFV >3.84) and *p* < 0.001 (ΔOFV >10.83) were performed in forward inclusion and backward elimination procedures, respectively. Moreover, the clinical meaning of parameters with a significant reduction of model variability in covariate selection was also considered. In the modeling process, the condition numbers were calculated to avoid over-parameterization, accepting no more than 1,000 as the criterion (Owen and Fiedler-Kelly, 2014).

#### 2.2.3 Implementation of Published Models

Published models were rebuilt and fixed parameters were reported in each study. Prediction- and simulation-based diagnostics were then used to evaluate the predictive performance of the published models (Zhao et al., 2016; Mao et al., 2018). If specific continuous covariates were missing, the median of the dataset or the model population was imputed. The data were assumed to be in the negative category (e.g., not receiving concomitant omeprazole) if categorical covariates were not available.

### 2.3 Model Evaluation

The predictability of published popPK models and the newly built model was evaluated by prediction- and simulation-based diagnostics. To compare the accuracy and precision of model predictive performance, prediction-based prediction error (PE, Eq. 1), median prediction error (MDPE), and median absolute prediction error (MAPE) were calculated and estimated (Sheiner and Beal, 1981).

$$\text{PE }\left(\text{\%}\right)=\left(\frac{\text{PRED}-\text{OBS}}{\text{OBS}}\right)\times 100$$ (1)

The percentages of PE within 20% (F_20_) and 30% (F_30_) were used as the combination index of both the accuracy and precision.

The goodness-of-fit plots were examined for model evaluation. The model stability and precision of parameter estimates were assessed using the bootstrap method (Ette et al., 2003). By random sampling with replacement in Perl modules, 2000 bootstrap datasets were generated (Ette, 1997). The final popPK model was compared with each of the bootstrap datasets to obtain 95% confidence intervals (CI) for all model parameters.

The predictability of the candidate model was evaluated using prediction-corrected visual predictive checks (pcVPCs) with 2000 simulations (Bergstrand et al., 2011). The 95% CI for the median, and the 5th and 95th percentiles of the simulations were calculated and compared with the observations, binning automatically.

### 2.4 Model Application

Using parameter estimates from the most suitable model, Monte Carlo simulations were performed. The objective of this study was to determine the influence of covariates on the incidence of delayed MTX elimination. The proportion of patients with MTX concentrations ≤0.2 μmol L^−1^ at 72 h was analyzed, as 3 g m^−2^ was administered for standard patients (with BSA 1.6 m^2^) with different covariate levels (2.5th percentile, median, and 97.5th percentile). After simulating 1,000 hypothetical individuals, the time-concentration profiles were obtained in each scenario.

## 3 Results

### 3.1 Patients and Data Collection

Patient demographic and physical characteristics are presented in Table 1. Data from 77 patients, covering 377 courses (1–17 per patient), and 1458 MTX plasma concentrations were available for analysis. The doses administered to patients ranged from 2 to 15.8 g. MTX dosage was transformed into molar equivalents by dividing them by the molecular weight (MTX: 222 g mol^−1^, http://chem.nlm.nih.gov/chemidplus/). For 92.3% of the treatment cycles, patients had short infusions, ranging from 1 to 4 h, whereas the remaining patients had long infusions, ranging up to 28.25 h.

In total, 567 concentrations were below the limit of quantification (LOQ), among which 236 were the second samples below the LOQ in the same treatment cycle. The M6 method was used to handle samples below the LOQ of 0.3 μmol L^−1^ as Gallais *et al.* done previously (Beal, 2001; Gallais et al., 2020). For concentrations under the lower LOQ (LLOQ), the first measurement in each continuous series was set to LLOQ/2, with the following measurements being treated as missing values. More complex approaches such as the M3/M4 method (likelihood estimation) did not improve model predictability, and thus were not evaluated further. The description of the sampling points and samples below the LOQ is provided in Supplementary Table S2.

### 3.2 Population Pharmacokinetic Models

#### 3.2.1 Review of Published popPK Studies on HD-MTX

Eight eligible popPK studies (Faltaos et al., 2006; Min et al., 2009; Simon et al., 2013; Benz-de Bretagne et al., 2014; Nader et al., 2017; Mei et al., 2018; Gallais et al., 2020; Yang et al., 2020) were identified during the literature review for further analysis. The screening process is presented in Text S1. The details of each study are summarized in Table 2. Among them, seven were single-center studies, whereas one was conducted at two centers (Benz-de Bretagne et al., 2014). Additionally, four studies were primarily conducted in France (Faltaos et al., 2006; Simon et al., 2013; Benz-de Bretagne et al., 2014; Gallais et al., 2020), three in China (Min et al., 2009; Mei et al., 2018; Yang et al., 2020), and one in Qatar (Nader et al., 2017). Furthermore, seven studies in the analysis had a small sample size of less than 1,000 concentrations (Faltaos et al., 2006; Min et al., 2009; Simon et al., 2013; Benz-de Bretagne et al., 2014; Nader et al., 2017; Mei et al., 2018; Yang et al., 2020). Four bioassay methods, HPLC, EMIT, TDx, and HEI, were used in seven studies (Table 2). Bioassay information was not provided in one study (Nader et al., 2017).

**TABLE 2:** Summary of published population pharmacokinetic studies of HD-MTX in adult patients with lymphoid malignancy.

Study (publication year)	Country (single/multiple sites)	Number of samples/Patients (M/F)	Dosage regimen	Sampling schedule	Bio-assay		PK parameters and formula	BSV% (IOV%)	Residual error	Evaluation
Faltaos et al. (2006)	France (Single)	496/51 (28/23)	1–8 g/m^2^,	C_24_/C_48_ and other two samples^a^	EMIT	CL/*F*	7.1×(AGE/62)^−0.22^ ×(SCR/67)^−0.43^	22.0 (16.5)	46.0%	GOF, Bootstrap, MPE,RMSE
			1–6 h, i.v.			V_c_/*F*	25.1	22.5	0.015 μmol/L	
						Q/*F*	0.15	51.0		
						V_p_/*F*	2.7	64.0		
Min et al. (2009)	China (Single)	400/82 (60/22)	1.5–9 g, 24 h, i.v.	Before and 6,12,18,24,30,36,44,50,56,68,74,80,92 h after infusion	TDx	CL/*F*	7.45×[1 + 0.224×(0.89-SCR/100)]	50.6	42.3%	GOF,
						V_c_/*F*	25.9×[1–0.00937×(66-WT)]	22.5	0.039 μmol/L	MPE,RMSE,
						Q/*F*	0.333	70.4		Cross-over validation
						V_p_/*F*	9.23	97.8		
Simon et al. (2013)	France (Single)	496/50 (27/23)	1–8 g/m^2^, 1–6 h, i.v.	At the end of infusion and 8–12,24,48, 72 h until <0.03 μmol/L	EMIT	CL/*F*	3.99×(1.63, if ABCC2 CT or TT) + 1.91×(CrCL/89)	28.7^d^	44.4%	GOF,
						V_1_/*F*	19.0×(1.63, if ABCC2 CT or TT)	36.7^d^		Bootstrap,
						Q_2_/*F*	0.1	/		VPC
						V_2_/*F* Q_3_/*F*	1.58	/		
						V_3_/*F*	0.021	/		
							1.99	/		
Bretagne *et al.* (2014)	France (Multiple)	363/81 (46/35)	1–8 g/m^2^,	C_24_/C_48_/C_72_, then q24 h until <0.2 μmol/L	EMIT (Paris)	CL/*F*	7.05×(CrCL/91.6)^0.27^×(DP3/0.6)^0.16^–0.93×SCO2	23.0	41.7%	GOF, Bootstrap, NPDE
			3–24 h, i.v.		TDx (Tours)	V_c_/*F*	23.5	34.0		
						Q/*F*	0.13	/		
						V_p_/*F*	3.01	32.1		
Nader et al. (2017)	Qatar (Single)	530/37 (31/6)	0.5–7 g/m^2^,	q12 h or q24 h until <0.05 μmol/L	NA	CL/*F*	15.7×(HCT/32)^0.85^	34.9 (47.4)^b^(31.1)^b^	33.4%	GOF,
			4–6 h or 24 h, i.v.			V_c_/*F*	79.2×(WT/69)^1.29^	/		VPC
						Q/*F*	0.97	/		
						V_p_/*F*	51.4	63.2		
Mei et al. (2018)	China (Single)	701/98 (53/45)	0.9–5.4 g/m^2^ 1.3–8.2 h, i.v.	C_24_/C_48_/C_72_/C_96_	HPLC	CL/*F*	6.67×(SCR/68.1)^−0.48^×(BSA/1.75)^1.17^	40.0	3.02 μmol/L	GOF,
						V_c_/*F*	24.46×(AGE/57.16)^0.81^	42.7		Bootstrap,
						Q/*F*	0.047	25.1		VPC
						V_p_/*F*	1.32	63.0		
Yang et al. (2020)	China (Single)	852/91 (64/27)	1–3 g/m^2^, i.v.	NA	HPLC	CL/*F*	6.03×(CrCL/115.1)^0.414^	51.6 (15.4)	0.32 μmol/L	GOF,
						V_c_/*F*	20.7	48.3		Bootstrap,
						Q/*F*	0.074×(BSA/1.65)^1.43^	65.6		VPC
						V_p_/*F*	3.76	/		
Gallais et al. (2020)	France (Single)	1,179/328 (180/133)^c^	1–8 g/m^2^, 0.5–36 h, i.v.	C_36_/C_48_, then q24 h until <0.2 μmol/L	HEI	CL/*F*	8.3×(AGE/50)^−0.317^	23.0 (22.0)	34.0%	GOF
					HPLC	V_c_/*F*	27.4	/		
						Q/*F*	0.15 (fixed)	/		
						V_p_/*F*	3.1×(WT/70)^0.453^	38.0		

ABCC2, -24 C > T SNP (rs717620) in 5′-UT of the ATP-binding cassette transporter; BSA, body surface area (m^2^); BSV, between subject variability; CL/*F*, apparent clearance (l h^−1^); C_*n*_, concentration at *n* hours post-dose; CrCL, creatinine clearance (ml min^−1^); DP3, the change of basal value of urinary coproporphyrin I/coproporphyrin I + III ratio at the time of hospital discharge refer to the MTX pre-administration; EMIT, enzyme multiplied immunoassay technique; F, female; FPIA, fluorescence polarization immunoassay; GOF, goodness-of-fit plots; HCT, hematocrit (%); HD-MTX, high dose-methotrexate; HEI, homogeneous enzyme immunoassay; IOV, inter-occasion variability; LC/MS, liquid chromatography/mass; M: male; MPE, median prediction error; NPDE, normalized prediction distribution error; PCNSL, primary central nervous system lymphoma; Q/*F*, apparent inter-compartmental clearance (l h^−1^); RMSE, root mean square error; SCO2, co-administered with at least one drug of score 2; SCR, serum creatinine (μmol L^−1^); TDx, FPIA using TDx^®^ analysers; V_c_/*F*, apparent volume of distribution of central compartment (l); V_p_/*F*, apparent volume of distribution peripheral compartment (l); VPC, visual predictive check; WT, bodyweight (kg).

a Two supplementary samples: at the end of infusion and between 8 and 12 h from the beginning of the infusion. Eventual follow up plasma level determination was done at 72 h, 96 h or more.

b IOV of 47.4 and 31.1% on MTX CL, for the second and third dosing occasions.

c 15 patients not included owing to missing toxicity information.

d Correlation is CL ∼ V_C_, 0.78.

The covariates of the published final CL/*F* models included age, SCr, CrCL, HCT, BSA, change in basal urinary coproporphyrin I/coproporphyrin I + III ratio value at the time of hospital discharge (MTX pre-administration [DP3]), co-administration with at least one drug of score 2 (SCO2), and ABCB2 genotype. SCr, CrCL, and age were the most frequently identified covariates in the final models and were reported in three, two, and two studies, respectively (Supplementary Table S3 and Supplementary Table S4). Body size was screened in all studies, and three included it in the volume of distribution, whereas one included it in CL/*F* and Q/*F*. Moreover, ABCC2 polymorphisms were screened in two studies, and it was included in the final model in one study (Simon et al., 2013).

#### 3.2.2 Population Pharmacokinetic Model Development

Data was described using a two-compartment PK structural model (ADVAN3, TRANS4 subroutine) with linear elimination. The exponential model provided the best result for the residual variability from the results of the OFVs and the distribution of residuals in the diagnostic plots. The parameter estimates and associated precisions of the base model are presented in Table 3.

**TABLE 3:** Parameter estimates for the base model, final model and bootstrap procedure.

Parameters	Base model		Final model			Bootstrap of final model	
	Estimate	RSE (%)	Estimate	RSE (%)	Shrinkage (%)	Median	95% CI
Objective function value	4306.6	/	3455.9	/	/	/	/
CL/*F* (L h^−1^)	4.8	4.3	4.91	3.7	/	4.97	4.37–5.44
V_c_/*F* (L)	20.9	5.6	18.4	3.8	/	18.0	16.5–20.3
Q/*F* (L h^−1^)	0.09	14.0	0.063	9.8	/	0.073	0.022–0.10
V_p_/*F* (L)	5.9	26.3	2.18	14.1	/	2.17	1.59–2.77
Covariate effect on CL/*F*							
CrCL	/	/	0.49	22.6	/	0.50	0.29–0.69
ALB	/	/	0.35	50.6	/	0.35	0.031–0.72
AGE	/	/	0.89	9.4	/	0.90	0.72–1.06
Between-subject variability							
CL/*F* (%)	39.2	24.9	20.9	25.5	25.5	20.3	1.5–29.5
V_c_/*F* (%)	36.9	40.4	19.6	26.9	20.9	20.4	17.3–32.8
Q/*F* (%)	62.3	18.3	40.6	18.1	19.4	36.7	18.7–54.3
V_p_/*F* (%)	44.2	14.4	30.4	17.5	47.7	29.2	13.8–40.7
Inter-occasion variability							
IOV on CL	/	/	24.7	20.4	43.8	24.2	5.6–35.4
Residual variability							
Proportional (%)	58.3	6.5	40.1	6.3	11.5	39.6	34.9–44.9

ALB, albumin; CI, percentile confidence intervals; CL/*F*, apparent clearance; CrCL, creatinine clearance; *F*, the bioavailability relative to 1; IOV, inter-occasion variability; Q/*F*, inter-compartmental clearance; RSE, relative standard error; V_c_/*F*, apparent central volume of distribution; V_p_/*F*, apparent peripheral volume of distribution; RSE, relative standard error.

Mechanistic plausibility was mainly considered as a potential covariate incorporated into the base model. As MTX is primarily eliminated unchanged by renal excretion, the effect of CrCL on MTX PK CL/*F* was tested first. The OFV substantially declined when CrCL was included exponentially (ΔOFV -97.6, *p* < 0.001), indicating a significant improvement in the model. A decrease (18.0%) in CL/*F* was observed as the CrCL decreased from 90 ml min^−1^ to 60 ml min^−1^.

The pathophysiological factors influencing MTX protein binding were then investigated empirically. ALB and HCT were included in the model to assess which was more suitable for describing the change in protein binding in the MTX PK process (ΔOFV -59.9 *vs.* -14.8, *p* < 0.001), and ALB was included in the final model. As 24.6% of patients were above 60 years old, age was also investigated by separating patients into two groups (i.e., older, or younger than 60 years). The CL/*F* of elderly patients (age >60) was 11.0% lower than that of the younger patients (ΔOFV -13.3, *p* < 0.001).

Moreover, the influence of morphological characteristics, such as body weight, BSA, and body mass index (BMI), on PK disposition parameters was tested based on allometric scaling theory (West et al., 1997; Anderson and Holford, 2009). However, no significant differences were observed. The presence of DIS ≥2 was added to the CL/*F* to test the influence of drug-drug interaction factors on the MTX PK process. However, the drop in the OFV was 3.6 and no significant differences were observed. The step-by-step covariate screening procedure is shown in Supplementary Table S5 and the correlation between BSVs is presented in Supplementary Figure S1.

The additional estimation of the IOV for CL, which was estimated to be 24.7%, significantly improved model predictions (ΔOFV -679.9, *p* < 0.001), which suggests that elimination parameters vary across MTX courses.

The parameters of the final model are listed in Table 3. The final model with CL/*F* covariates was described as follows.

$$\text{CL}/F=4\text{.}91\text{×}{\left(\text{CrCL}/98\right)}^{0.49}\text{×}{\left(\text{ALB}/40\right)}^{\text{0}\text{.}35}\text{×}\left(\text{0}\text{.}89\text{, if age > }60\right)$$

In the final model, all retained covariates caused a significant increase in OFV upon removal. The condition number of the final model was 83.6. Shrinkage analysis for CL showed a mean η-CL shrinkage of 25.5% and ε-shrinkage of 24.9%.

### 3.3 Model Evaluation

The predictive performance of published models was evaluated in the evaluation population dataset using the prediction- and simulation-based diagnostics described above. Supplementary Figure S2 shows the goodness-of-fit plots for all published models. The values of bias and inaccuracies of the published models are presented in Table 4. All models presented an underestimation of concentrations with large inaccuracies. The predictive performance of the predicted HD-MTX concentration-time profiles, as revealed by the pcVPC of the published models, was highly variable (Supplementary Figure S3). None of the models presented acceptable bias or inaccuracy values.

**TABLE 4:** Results of the prediction-based metrics.

Models	MDPE	MAPE	F_20_	F_30_
**Published models**				
Faltaos et al.(2006)	−35.4	47.7	18.5	27.4
Min et al. (2009)	−29.9	43.0	20.4	31.7
Simon et al. (2013)	−53.1	54.2	15.6	25.1
Bretagne et al. (2014)	−27.9	40.2	23.5	36.5
Nader et al. (2017)	−68.4	70.6	11.0	17.0
Mei et al. (2018)	−32.7	50.5	19.2	29.9
Yang et al. (2020)	−28.8	37.0	26.3	41.3
Gallais et al. (2020)	−45.7	47.6	16.0	28.3
**Newly built model**				
Final model	−10.2	36.4	29.7	42.3

F_20_, the percentages of prediction errors within 20%; F_30_, the percentages of prediction errors within 30%; MAPE, median absolute prediction error; MDPE, median prediction error.

**FIGURE 1:**
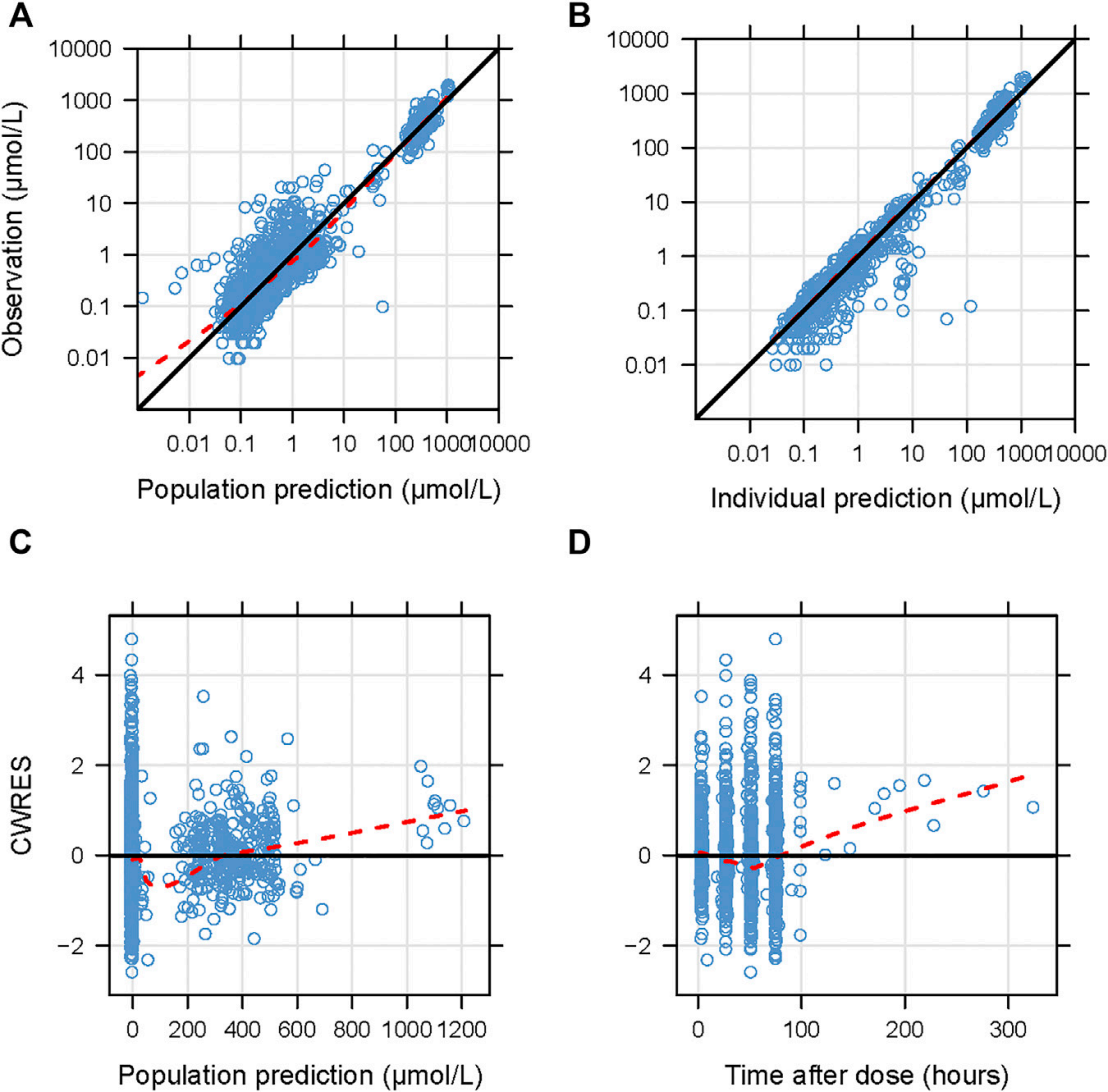
Diagnostic goodness-of-fit plots for the final model. **(A)** Observations versus population predictions; **(B)** observations versus individual predictions; **(C)** conditional weighted residuals (CWRES) versus population predictions; **(D)** CWRES versus time after dose. **(A–D)** The locally weighted regression line (*red dashed lines*). **(A,B)** the line of unity (*black solid lines*), and **(C,D)** y = 0 (*solid lines*) are shown.

**FIGURE 2:**
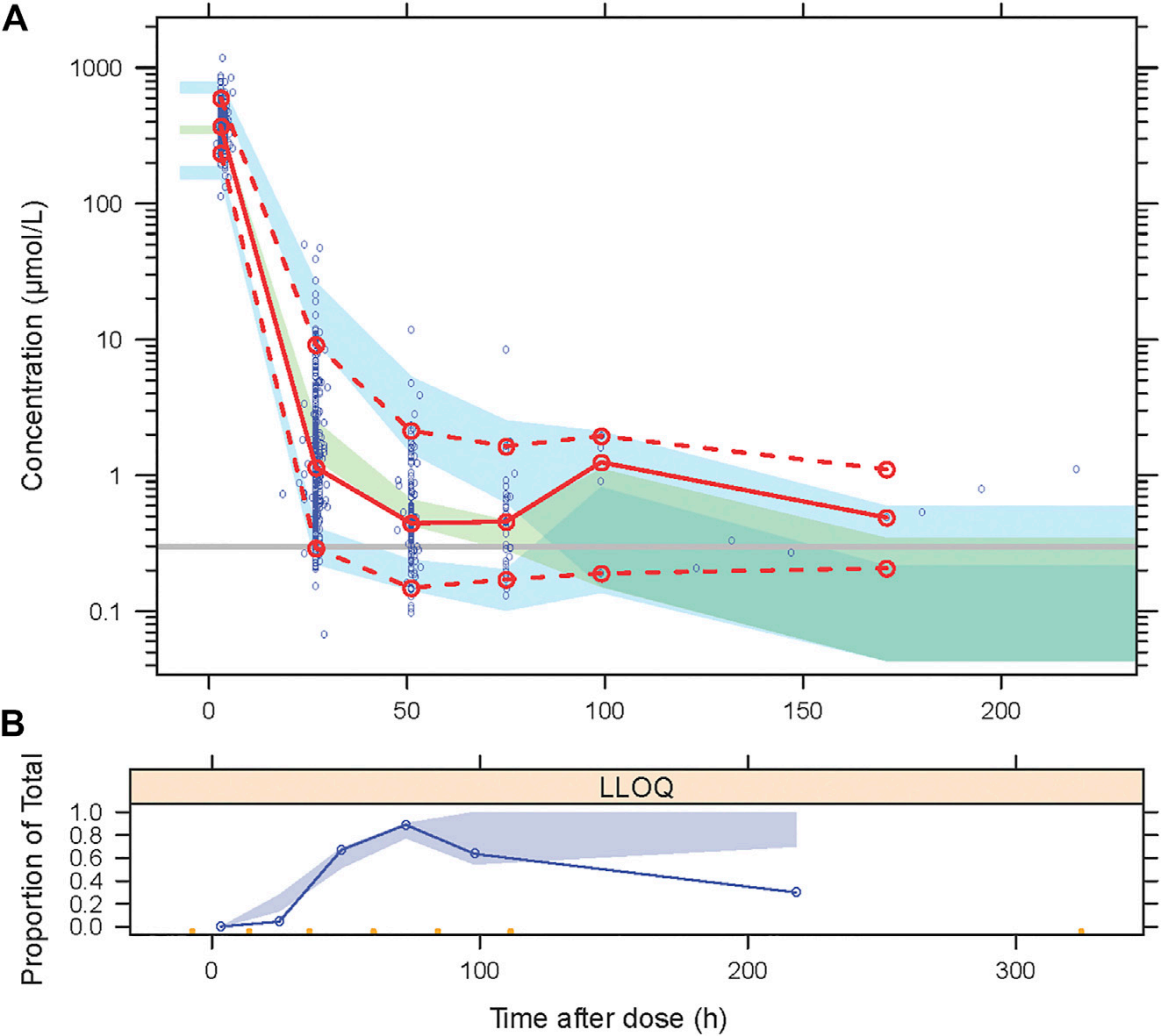
Visual predictive checks (VPCs) for the final model, based on 2000 simulations. **(A)** The *red solid line* connects median observed values per bin, the *red dashed lines* connect the 5th and 95th percentiles of the observations. The *blue areas* represent the 95% confidence interval of the 5th and 95th percentiles. The *green area* indicates the confidence interval of the median. The *Y*-axis is shown in a logarithmic scale. **(B)** Open circles represent the observed fraction of censored data, and the shaded area represents the 95% confidence interval of the simulated fraction of censored data.

The goodness-of-fit plots for the newly built model are presented in Figure 1 showing no structural bias. The pcVPCs of the final model are depicted in Figure 2. The data below the LOQ were included in the model evaluation to assess the capability of the final model to describe these points. The simulated data corresponded well with the observed data, except for data below the LOQ around 24 h and more than 100 h after the dose. However, as only 18 samples and 10 samples below the LOQ in these periods were included in our study, there were no significant model misspecifications. Model stability was also confirmed with consistent bootstrapped parameter estimates differing by no more than 15% from its corresponding estimate in the final model (Table 3). The MDPE and MAPE were -10.2% and 36.4%, respectively. The relatively low values of MDPE and MAPE further confirmed the high prediction accuracy of the final model.

### 3.4 Model Application

**FIGURE 3:**
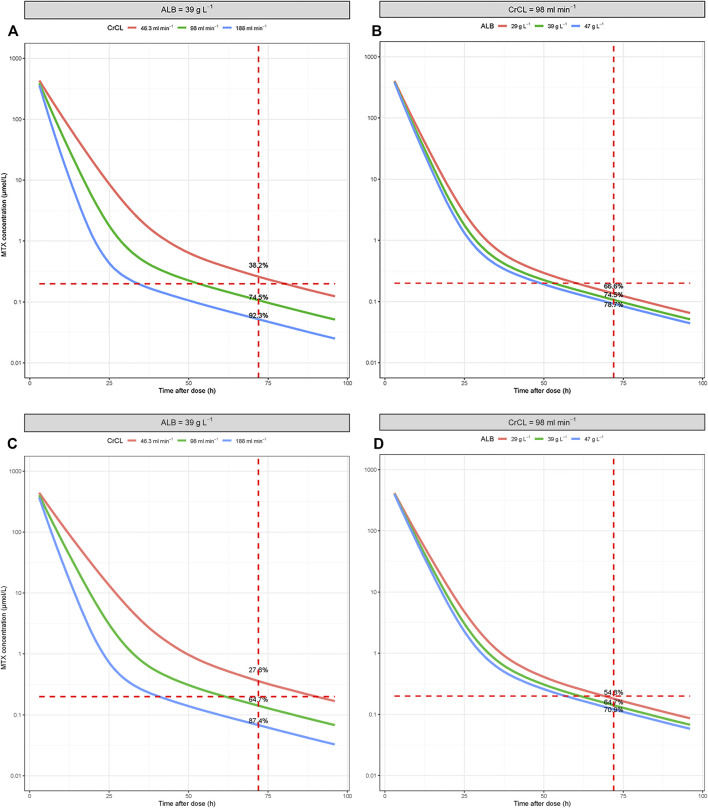
Simulated time-concentration profiles of MTX under conditions involving different covariate levels. **(A,B)** For patients ≤60 years old; **(C,D)** for patients >60 years old.

The characteristics of the involved covariates in the simulation are listed in Supplementary Table S6. The results of the Monte Carlo simulation are presented in Supplementary Table S7. The predicted time course of MTX concentration in the scenarios with median covariate levels is presented in Figure 3. Based on the simulation, elderly patients with renal dysfunction and hypoalbuminemia have a higher incidence of delayed MTX elimination. The percentage decreased from 64.7% to 27.6% when the CrCL decreased from 98 ml min^−1^ to 46.3 ml min^−1^, and the percentage decreased from 64.7% to 54.8% when ALB decreased from 39 g L^−1^ to 29 g L^−1^.

## 4 Discussion

To the best of our knowledge, this is the first comprehensive analysis of the predictability of published HD-MTX popPK models. Prediction-based metrics and simulation-based diagnostics were applied to assess the accuracy and precision of the published models. According to our results, the published models were insufficient for cross-center use. Thus, constructing a new popPK model based on an independent dataset is a priority.

Whether the assumed description of the popPK model is in agreement with the population it is intended to treat, depends on two aspects: a well-developed model with reliable a *priori* PK parameter distribution (mixed and random) and center-related factors that may result in unexplained inter-study variability (Laporte-Simitsidis et al., 2000). The center-related factors, such as study design, ethnic differences, assay methods, and modeling strategies, may influence model predictability (Mao et al., 2018; Mao et al., 2020). In this study, the inclusion of older patients with primary central nervous system lymphoma, as well as inconsistencies in the assay methods, may be the primary causes of the poor cross-center predictability observed.

Although the published models were inadequate for cross-center use, the information included in these models can be utilized to guide the building of new models. In previous studies, we found that theory-based modeling is helpful to improve model predictability (Mao et al., 2020; Mao et al., 2021). Unlike empirical stepwise covariate selection, theory-based covariate selection allows the incorporation of relationships linking parameters and covariates based on a fundamental understanding of PK processes rather than only on the available data, and may improve model predictability (Danhof et al., 2008). Therefore, we conducted modeling based on the combination of theoretical mechanisms and data properties in this study.

According to published studies, CrCL was the most frequently identified covariate. This was consistent with the PK characteristics of MTX. MTX and its major metabolite, 7-OH-MTX, are mainly eliminated by glomerular filtration and active secretion (Shitara et al., 2006). After analyzing HD-MTX serum samples and urine samples, it was revealed that MTX renal clearance is 83% of the total clearance and CrCL, as an index of glomerular filtration rate, has a great effect on renal clearance but not on non-renal clearance (Fukuhara et al., 2008). In the present study, as no urine samples were included, the influence of CrCL was added to the total clearance. As the CrCL doubling decreased from 120 ml min^−1^ to 60 ml min^−1^, the CL/*F* decreased by 28.8%, which is consistent with the results obtained using published models (17.1%–50%), and in patients with CrCL increase from 120 ml min^−1^–180 ml min^−1^, the CL/*F* increased by 22.0%, which is close to the results obtained using published models (11.6%–19.6%) (Simon et al., 2013; Benz-de Bretagne et al., 2014; Yang et al., 2020).

Alterations in HCT or hemoglobin may affect MTX binding and thus influence the PK process. Hypoalbuminemia is reportedly associated with a significantly increased time for MTX clearance (Reiss et al., 2016). Therefore, the reduction of ALB is a risk factor for delayed MTX elimination in HD-MTX monotherapy (Kataoka et al., 2021). In the final model, a reduction in ALB from 50.0 g L^−1^ to 24.0 g L^−1^ may result in a 22.7% decrease in MTX CL/*F*. The change in MTX CL/*F* may have clinical effects on MTX exposure and treatment response. Moreover, malignant cachexia or liver metastases induced by hypoalbuminemia may increase the half-life of MTX PK, which may be associated with unanticipated toxicity (Li and Gwilt, 2002).

The CL/*F* was decreased by 11.0% in elderly patients, which may also be a risk factor for delayed elimination. The incidence of delayed clearance of MTX is higher in older patients and during the first cycle of treatment (Bacci et al., 2003). After comparing the PK of total and free MTX in rheumatoid arthritis patients, it has been shown that elderly patients have a longer elimination half-life of free and total MTX (Bressolle et al., 1997). For elderly patients, altered metabolic functions are a result of the complex processes of aging-related physiological changes in the functional reserve of multiple systems and organs. Furthermore, renal excretion is reduced (up to 50%) in approximately two-thirds of elderly patients, which can potentially cause the delayed elimination of MTX (Klotz, 2009).

The estimated PK parameters in our final model were consistent with those in previous reports, except that the CL/*F* was lower than that in published studies. This phenomenon may be owing to the influence of drug-drug interactions. In our study, the proportion of samples with DIS ≥2 was more than 85.4%. Co-administration, such as that of non-steroidal anti-inflammatory drugs, β-lactamins, and proton pump inhibitors, has been reported to be an important factor that influences MTX elimination (Iven and Brasch, 1990; Suzuki et al., 2009; Kawase et al., 2016), and may inhibit the excretion of MTX. A reduction in CL/*F*, by 0.929, as at least one score 2 drug was used (Benz-de Bretagne et al., 2014).

Previous studies have demonstrated that variability exists between MTX therapy cycles (Fukuhara et al., 2008; Min et al., 2009; Kim et al., 2012; Mei et al., 2018). In this study, we estimated course-to-course variability with the inclusion of IOV terms in the random effects model. IOV may arise from variable renal function owing to repeated chemotherapy, drug-drug interactions, or other environmental factors (Breedveld et al., 2004; El-Sheikh et al., 2007; Leveque et al., 2011).

No gene information was available for analysis in this study. The influence of polymorphisms in genes encoding transporters or enzymes during the MTX PK process was inconsistent between different studies, which may partly be accounted for by the different types of patients. The sample size did not have sufficient power to detect a significant association between the target single nucleotide polymorphisms (SNPs) and the MTX PK properties, and the different frequencies of SNPs owing to racial differences (Simon et al., 2013; Lui et al., 2018; Yang et al., 2020). We will consider this point in a future study.

In relative terms, body size and composition were less influential in the model of MTX CL (Pai et al., 2020). A statistically significant reduction in the IIV of CL in only 15% of anticancer drugs is associated with BSA. Moreover, the relative reduction in the variability of CL is between 15% and 35% (Baker et al., 2002). Among the eight published models, only one model included the influence of body size on CL. It is also worth mentioning that body size does not take into account abnormal body habitus such as cachexia or morbid obesity (Gurney and Shaw, 2007; Gallais et al., 2020). In our study, only two patients had a BMI ≥30. Therefore, body size was not a risk factor identified in this study.

Certain limitations were noted in our study. First, this study relied on routine MTX monitoring data that was retrospectively collected and did not include information regarding aspects of supportive care, such as fluid hydration and urine pH, which may have impacted MTX CL and influenced model predictability. Second, MTX concentrations were measured using multiple analytical methods between studies. No conversion of immunoassays may introduce uncertainty into the external evaluation results. Third, no information on MTX metabolites was available for this study. The inclusion of MTX metabolite information in the popPK model could provide more insights into MTX disposal. Therefore, this should be the focus of future studies.

Identifying ways to obviate MTX delays could facilitate toxicity prediction, rational dose adjustments, and theoretically improve treatment outcomes (Crews et al., 2004). According to our results, the currently published models are not sufficiently reliable for cross-center use; the newly built model provides better predictability. The elderly patients and those with renal dysfunction, hypoalbuminemia are at higher risk of delayed elimination.

## Data Availability

The raw data supporting the conclusions of this article will be made available by the authors, without undue reservation.

## References

[B1] Bacci G. Ferrari S. Longhi A. Forni C. Loro L. Beghelli C. (2003). Delayed Methotrexate Clearance in Osteosarcoma Patients Treated with Multiagent Regimens of Neoadjuvant Chemotherapy. Oncol. Rep. 10 (4), 851–857. 10.3892/or.10.4.851 12792734

[B2] Baker S. D. Verweij J. Rowinsky E. K. Donehower R. C. Schellens J. H. Grochow L. B. (2002). Role of Body Surface Area in Dosing of Investigational Anticancer Agents in Adults, 1991-2001. J. Natl. Cancer Inst. 94 (24), 1883–1888. 10.1093/jnci/94.24.1883 12488482

[B3] Baram J. Allegra C. J. Fine R. L. Chabner B. A. (1987). Effect of Methotrexate on Intracellular Folate Pools in Purified Myeloid Precursor Cells from normal Human Bone Marrow. J. Clin. Invest. 79 (3), 692–697. 10.1172/JCI112872 3818945 PMC424178

[B4] Beal S. L. (2001). Ways to Fit a PK Model with Some Data below the Quantification Limit. J. Pharmacokinet. Pharmacodyn 28 (5), 481–504. 10.1023/a:1012299115260 11768292

[B5] Beal S. L. Sheiner L. B. Boeckmann A. Bauer R. J. (1989). NONMEM User's Guides. Ellicott City, MD, USA: Icon Development Solutions.

[B6] Benz-de Bretagne I. Zahr N. Le Gouge A. Hulot J. S. Houillier C. Hoang-Xuan K. (2014). Urinary Coproporphyrin I/(I + III) Ratio as a Surrogate for MRP2 or Other Transporter Activities Involved in Methotrexate Clearance. Br. J. Clin. Pharmacol. 78 (2), 329–342. 10.1111/bcp.12326 24433481 PMC4137825

[B7] Bergstrand M. Hooker A. C. Wallin J. E. Karlsson M. O. (2011). Prediction-corrected Visual Predictive Checks for Diagnosing Nonlinear Mixed-Effects Models. AAPS J. 13 (2), 143–151. 10.1208/s12248-011-9255-z 21302010 PMC3085712

[B8] Breedveld P. Zelcer N. Pluim D. Sönmezer O. Tibben M. M. Beijnen J. H. (2004). Mechanism of the Pharmacokinetic Interaction between Methotrexate and Benzimidazoles: Potential Role for Breast Cancer Resistance Protein in Clinical Drug-Drug Interactions. Cancer Res. 64 (16), 5804–5811. 10.1158/0008-5472.CAN-03-4062 15313923

[B9] Bressolle F. Bologna C. Kinowski J. M. Arcos B. Sany J. Combe B. (1997). Total and Free Methotrexate Pharmacokinetics in Elderly Patients with Rheumatoid Arthritis. A Comparison with Young Patients. J. Rheumatol. 24 (10), 1903–1909. 9330930

[B10] Cheng Y. Wang C. Y. Li Z. R. Pan Y. Liu M. B. Jiao Z. (2020). Can Population Pharmacokinetics of Antibiotics Be Extrapolated? Implications of External Evaluations. Clin. Pharmacokinet. 60, 53–68. 10.1007/s40262-020-00937-4 32960439

[B11] Cockcroft D. W. Gault M. H. (1976). Prediction of Creatinine Clearance from Serum Creatinine. Nephron 16 (1), 31–41. 10.1159/000180580 1244564

[B12] Crews K. R. Liu T. Rodriguez-Galindo C. Tan M. Meyer W. H. Panetta J. C. (2004). High-dose Methotrexate Pharmacokinetics and Outcome of Children and Young Adults with Osteosarcoma. Cancer 100 (8), 1724–1733. 10.1002/cncr.20152 15073863

[B13] Csordas K. Hegyi M. Eipel O. T. Muller J. Erdelyi D. J. Kovacs G. T. (2013). Comparison of Pharmacokinetics and Toxicity after High-Dose Methotrexate Treatments in Children with Acute Lymphoblastic Leukemia. Anticancer Drugs 24 (2), 189–197. 10.1097/CAD.0b013e32835b8662 23187460

[B14] Danhof M. de Lange E. C. Della Pasqua O. E. Ploeger B. A. Voskuyl R. A. (2008). Mechanism-based Pharmacokinetic-Pharmacodynamic (PK-PD) Modeling in Translational Drug Research. Trends Pharmacol. Sci. 29 (4), 186–191. 10.1016/j.tips.2008.01.007 18353445

[B15] Dupuis C. Mercier C. Yang C. Monjanel-Mouterde S. Ciccolini J. Fanciullino R. (2008). High-dose Methotrexate in Adults with Osteosarcoma: a Population Pharmacokinetics Study and Validation of a New Limited Sampling Strategy. Anticancer Drugs 19 (3), 267–273. 10.1097/cad.0b013e3282f21376 18510172

[B16] El-Sheikh A. A. van den Heuvel J. J. Koenderink J. B. Russel F. G. (2007). Interaction of Nonsteroidal Anti-inflammatory Drugs with Multidrug Resistance Protein (MRP) 2/ABCC2- and MRP4/ABCC4-Mediated Methotrexate Transport. J. Pharmacol. Exp. Ther. 320 (1), 229–235. 10.1124/jpet.106.110379 17005917

[B17] Ette E. I. (1997). Stability and Performance of a Population Pharmacokinetic Model. J. Clin. Pharmacol. 37 (6), 486–495. 10.1002/j.1552-4604.1997.tb04326.x 9208355

[B18] Ette E. I. Williams P. J. Kim Y. H. Lane J. R. Liu M. J. Capparelli E. V. (2003). Model Appropriateness and Population Pharmacokinetic Modeling. J. Clin. Pharmacol. 43 (6), 610–623. 10.1177/0091270003253624 12817524

[B19] Ette E. I. Williams P. J. (2004). Population Pharmacokinetics I: Background, Concepts, and Models. Ann. Pharmacother. 38 (10), 1702–1706. 10.1345/aph.1D374 15328391

[B20] Evans W. E. Crom W. R. Abromowitch M. Dodge R. Look A. T. Bowman W. P. (1986). Clinical Pharmacodynamics of High-Dose Methotrexate in Acute Lymphocytic Leukemia. Identification of a Relation between Concentration and Effect. N. Engl. J. Med. 314 (8), 471–477. 10.1056/NEJM198602203140803 3456079

[B21] Evans W. E. Relling M. V. Rodman J. H. Crom W. R. Boyett J. M. Pui C. H. (1998). Conventional Compared with Individualized Chemotherapy for Childhood Acute Lymphoblastic Leukemia. N. Engl. J. Med. 338 (8), 499–505. 10.1056/NEJM199802193380803 9468466

[B22] Faltaos D. W. Hulot J. S. Urien S. Morel V. Kaloshi G. Fernandez C. (2006). Population Pharmacokinetic Study of Methotrexate in Patients with Lymphoid Malignancy. Cancer Chemother. Pharmacol. 58 (5), 626–633. 10.1007/s00280-006-0202-0 16528531

[B23] Fukuhara K. Ikawa K. Morikawa N. Kumagai K. (2008). Population Pharmacokinetics of High-Dose Methotrexate in Japanese Adult Patients with Malignancies: a Concurrent Analysis of the Serum and Urine Concentration Data. J. Clin. Pharm. Ther. 33 (6), 677–684. 10.1111/j.1365-2710.2008.00966.x 19138246

[B24] Gallais F. Oberic L. Faguer S. Tavitian S. Lafont T. Marsili S. (2020). Body Surface Area Dosing of High-Dose Methotrexate Should Be Reconsidered, Particularly in Overweight, Adult Patients. Ther. Drug Monit. 43, 408–415. 10.1097/FTD.0000000000000813 32925658

[B25] Griggs J. J. Mangu P. B. Anderson H. Balaban E. P. Dignam J. J. Hryniuk W. M. (2012). Appropriate Chemotherapy Dosing for Obese Adult Patients with Cancer: American Society of Clinical Oncology Clinical Practice Guideline. J. Clin. Oncol. 30 (13), 1553–1561. 10.1200/JCO.2011.39.9436 22473167

[B26] Gurney H. Shaw R. (2007). Obesity in Dose Calculation: a Mouse or an Elephant? J. Clin. Oncol. 25 (30), 4703–4704. 10.1200/JCO.2007.13.1078 17947715

[B27] Hospira (2017). Label for Methotrexate Injection. Lake Forest, IL: Hospira. Available at: *https://www.accessdata.fda.gov/drugsatfda_docs/label/2011/011719s117lbl.pdf* (Accessed Dec 29, 2019).

[B28] Howard S. C. McCormick J. Pui C. H. Buddington R. K. Harvey R. D. (2016). Preventing and Managing Toxicities of High-Dose Methotrexate. Oncologist 21 (12), 1471–1482. 10.1634/theoncologist.2015-0164 27496039 PMC5153332

[B29] Huang C. Xia F. Xue L. Liu L. Bian Y. Jin Z. (2020). Coadministration of Vindesine with High-Dose Methotrexate Therapy Increases Acute Kidney Injury via BCRP, MRP2, and OAT1/OAT3. Cancer Chemother. Pharmacol. 85 (2), 433–441. 10.1007/s00280-019-03972-6 31691080

[B30] Iven H. Brasch H. (1990). Cephalosporins Increase the Renal Clearance of Methotrexate and 7-hydroxymethotrexate in Rabbits. Cancer Chemother. Pharmacol. 26 (2), 139–143. 10.1007/BF02897260 2189590

[B31] Karlsson M. O. Sheiner L. B. (1993). The Importance of Modeling Interoccasion Variability in Population Pharmacokinetic Analyses. J. Pharmacokinet. Biopharm. 21 (6), 735–750. 10.1007/BF01113502 8138894

[B32] Kataoka T. Sakurashita H. Kajikawa K. Saeki Y. Taogoshi T. Matsuo H. (2021). Low Serum Albumin Level Is a Risk Factor for Delayed Methotrexate Elimination in High-Dose Methotrexate Treatment. Ann. Pharmacother. 55, 1195–1202. 10.1177/1060028021992767 33543634

[B33] Kawase A. Yamamoto T. Egashira S. Iwaki M. (2016). Stereoselective Inhibition of Methotrexate Excretion by Glucuronides of Nonsteroidal Anti-inflammatory Drugs via Multidrug Resistance Proteins 2 and 4. J. Pharmacol. Exp. Ther. 356 (2), 366–374. 10.1124/jpet.115.229104 26659924

[B34] Keizer R. J. Karlsson M. O. Hooker A. (2013). Modeling and Simulation Workbench for NONMEM: Tutorial on Pirana, PsN, and Xpose. CPT Pharmacometrics Syst. Pharmacol. 2, e50. 10.1038/psp.2013.24 23836189 PMC3697037

[B35] Kim I. W. Yun H. Y. Choi B. Han N. Park S. Y. Lee E. S. (2012). ABCB1 C3435T Genetic Polymorphism on Population Pharmacokinetics of Methotrexate after Hematopoietic Stem Cell Transplantation in Korean Patients: a Prospective Analysis. Clin. Ther. 34 (8), 1816–1826. 10.1016/j.clinthera.2012.06.022 22796246

[B36] Klotz U. (2009). Pharmacokinetics and Drug Metabolism in the Elderly. Drug Metab. Rev. 41 (2), 67–76. 10.1080/03602530902722679 19514965

[B37] Laporte-Simitsidis S. Girard P. Mismetti P. Chabaud S. Decousus H. Boissel J. P. (2000). Inter-study Variability in Population Pharmacokinetic Meta-Analysis: when and How to Estimate it? J. Pharm. 89 (2), 155–167. 10.1002/(SICI)1520-6017(200002)89:2<155::AID-JPS3>3.0.CO;2-2 10688745

[B38] Levêque D. Santucci R. Gourieux B. Herbrecht R. (2011). Pharmacokinetic Drug-Drug Interactions with Methotrexate in Oncology. Expert Rev. Clin. Pharmacol. 4 (6), 743–750. 10.1586/ecp.11.57 22111860

[B39] Li J. Gwilt P. (2002). The Effect of Malignant Effusions on Methotrexate Disposition. Cancer Chemother. Pharmacol. 50 (5), 373–382. 10.1007/s00280-002-0512-9 12439595

[B40] Lui G. Treluyer J. M. Fresneau B. Piperno-Neumann S. Gaspar N. Corradini N. (2018). A Pharmacokinetic and Pharmacogenetic Analysis of Osteosarcoma Patients Treated with High-Dose Methotrexate: Data from the OS2006/Sarcoma-09 Trial. J. Clin. Pharmacol. 58 (12), 1541–1549. 10.1002/jcph.1252 29791011

[B41] Maia M. B. Saivin S. Chatelut E. Malmary M. F. Houin G. (1996). *In Vitro* and *In Vivo* Protein Binding of Methotrexate Assessed by Microdialysis. Int. J. Clin. Pharmacol. Ther. 34 (8), 335–341. 8864795

[B42] Mao J. Jiao Z. Qiu X. Zhang M. Zhong M. (2020). Incorporating Nonlinear Kinetics to Improve Predictive Performance of Population Pharmacokinetic Models for Ciclosporin in Adult Renal Transplant Recipients: A Comparison of Modelling Strategies. Eur. J. Pharm. Sci. 153, 105471. 10.1016/j.ejps.2020.105471 32682934

[B43] Mao J. Qiu X. Qin W. Xu L. Zhang M. Zhong M. (2021). Factors Affecting Time-Varying Clearance of Cyclosporine in Adult Renal Transplant Recipients: A Population Pharmacokinetic Perspective. Pharm. Res. 38 (11), 1873–1887. 10.1007/s11095-021-03114-9 34750720

[B44] Mao J. J. Jiao Z. Yun H. Y. Zhao C. Y. Chen H. C. Qiu X. Y. (2018). External Evaluation of Population Pharmacokinetic Models for Ciclosporin in Adult Renal Transplant Recipients. Br. J. Clin. Pharmacol. 84 (1), 153–171. 10.1111/bcp.13431 28891596 PMC5736841

[B45] Mei S. Li X. Jiang X. Yu K. Lin S. Zhao Z. (2018). Population Pharmacokinetics of High-Dose Methotrexate in Patients with Primary Central Nervous System Lymphoma. J. Pharm. Sci. 107 (5), 1454–1460. 10.1016/j.xphs.2018.01.004 29331383

[B46] Min Y. Qiang F. Peng L. Zhu Z. (2009). High Dose Methotrexate Population Pharmacokinetics and Bayesian Estimation in Patients with Lymphoid Malignancy. Biopharm. Drug Dispos 30 (8), 437–447. 10.1002/bdd.678 19746402

[B47] Moher D. Shamseer L. Clarke M. Ghersi D. Liberati A. Petticrew M. (2015). Preferred Reporting Items for Systematic Review and Meta-Analysis Protocols (PRISMA-P) 2015 Statement. Syst. Rev. 4, 1. 10.1186/2046-4053-4-1 25554246 PMC4320440

[B48] Nader A. Zahran N. Alshammaa A. Altaweel H. Kassem N. Wilby K. J. (2017). Population Pharmacokinetics of Intravenous Methotrexate in Patients with Hematological Malignancies: Utilization of Routine Clinical Monitoring Parameters. Eur. J. Drug Metab. Pharmacokinet. 42 (2), 221–228. 10.1007/s13318-016-0338-1 27059845

[B49] Owen J. s. Fiedler-Kelly J. (2014). Introduction to Population Pharmacokinetic/pharmacodynamic Analysis with Nonlinear Mixed Effects Models. Hoboken, New Jersey, USA: John Wiley & Sons. 10.1002/9781118784860 PMC5684957 25531560

[B50] Paci A. Veal G. Bardin C. Levêque D. Widmer N. Beijnen J. (2014). Review of Therapeutic Drug Monitoring of Anticancer Drugs Part 1--cytotoxics. Eur. J. Cancer 50 (12), 2010–2019. 10.1016/j.ejca.2014.04.014 24889915

[B51] Pai M. P. Debacker K. C. Derstine B. Sullivan J. Su G. L. Wang S. C. (2020). Comparison of Body Size, Morphomics, and Kidney Function as Covariates of High-Dose Methotrexate Clearance in Obese Adults with Primary Central Nervous System Lymphoma. Pharmacotherapy 40 (4), 308–319. 10.1002/phar.2379 32090349 PMC8855476

[B52] Plotkin S. R. Betensky R. A. Hochberg F. H. Grossman S. A. Lesser G. J. Nabors L. B. (2004). Treatment of Relapsed central Nervous System Lymphoma with High-Dose Methotrexate. Clin. Cancer Res. 10 (17), 5643–5646. 10.1158/1078-0432.CCR-04-0159 15355887

[B53] Ramsey L. B. Balis F. M. O'Brien M. M. Schmiegelow K. Pauley J. L. Bleyer A. (2018). Consensus Guideline for Use of Glucarpidase in Patients with High-Dose Methotrexate Induced Acute Kidney Injury and Delayed Methotrexate Clearance. Oncologist 23 (1), 52–61. 10.1634/theoncologist.2017-0243 29079637 PMC5759822

[B54] Reiss S. N. Buie L. W. Adel N. Goldman D. A. Devlin S. M. Douer D. (2016). Hypoalbuminemia Is Significantly Associated with Increased Clearance Time of High Dose Methotrexate in Patients Being Treated for Lymphoma or Leukemia. Ann. Hematol. 95 (12), 2009–2015. 10.1007/s00277-016-2795-7 27542957 PMC5572815

[B55] Reiter A. Schrappe M. Tiemann M. Ludwig W. D. Yakisan E. Zimmermann M. (1999). Improved Treatment Results in Childhood B-Cell Neoplasms with Tailored Intensification of Therapy: A Report of the Berlin-Frankfurt-Münster Group Trial NHL-BFM 90. Blood 94 (10), 3294–3306. 10552938

[B56] Sakura T. Hayakawa F. Sugiura I. Murayama T. Imai K. Usui N. (2018). High-dose Methotrexate Therapy Significantly Improved Survival of Adult Acute Lymphoblastic Leukemia: a Phase III Study by JALSG. Leukemia 32 (3), 626–632. 10.1038/leu.2017.283 28914260

[B57] Sheiner L. B. Beal S. L. (1981). Some Suggestions for Measuring Predictive Performance. J. Pharmacokinet. Biopharm. 9 (4), 503–512. 10.1007/BF01060893 7310648

[B58] Shitara Y. Horie T. Sugiyama Y. (2006). Transporters as a Determinant of Drug Clearance and Tissue Distribution. Eur. J. Pharm. Sci. 27 (5), 425–446. 10.1016/j.ejps.2005.12.003 16488580

[B59] Simon N. Marsot A. Villard E. Choquet S. Khe H. X. Zahr N. (2013). Impact of ABCC2 Polymorphisms on High-Dose Methotrexate Pharmacokinetics in Patients with Lymphoid Malignancy. Pharmacogenomics J. 13 (6), 507–513. 10.1038/tpj.2012.37 23069858

[B60] Suzuki K. Doki K. Homma M. Tamaki H. Hori S. Ohtani H. (2009). Co-administration of Proton Pump Inhibitors Delays Elimination of Plasma Methotrexate in High-Dose Methotrexate Therapy. Br. J. Clin. Pharmacol. 67 (1), 44–49. 10.1111/j.1365-2125.2008.03303.x 19076159 PMC2668083

[B61] Wall A. M. Gajjar A. Link A. Mahmoud H. Pui C. H. Relling M. V. (2000). Individualized Methotrexate Dosing in Children with Relapsed Acute Lymphoblastic Leukemia. Leukemia 14 (2), 221–225. 10.1038/sj.leu.2401673 10673736

[B62] West G. B. Brown J. H. Enquist B. J. (1997). A General Model for the Origin of Allometric Scaling Laws in Biology. Science 276 (5309), 122–126. 10.1126/science.276.5309.122 9082983

[B63] Widemann B. C. Adamson P. C. (2006). Understanding and Managing Methotrexate Nephrotoxicity. Oncologist 11 (6), 694–703. 10.1634/theoncologist.11-6-694 16794248

[B64] Yang L. Wu H. de Winter B. C. M. Sheng C. C. Qiu H. Q. Cheng Y. (2020). Pharmacokinetics and Pharmacogenetics of High-Dose Methotrexate in Chinese Adult Patients with Non-hodgkin Lymphoma: a Population Analysis. Cancer Chemother. Pharmacol. 85 (5), 881–897. 10.1007/s00280-020-04058-4 32246190

[B65] Zhao C. Y. Jiao Z. Mao J. J. Qiu X. Y. (2016). External Evaluation of Published Population Pharmacokinetic Models of Tacrolimus in Adult Renal Transplant Recipients. Br. J. Clin. Pharmacol. 81 (5), 891–907. 10.1111/bcp.12830 26574188 PMC4834594

[B66] Zhu J. J. Gerstner E. R. Engler D. A. Mrugala M. M. Nugent W. Nierenberg K. (2009). High-dose Methotrexate for Elderly Patients with Primary CNS Lymphoma. Neuro Oncol. 11 (2), 211–215. 10.1215/15228517-2008-067 18757775 PMC2718993

