# Supplementary material for: Evaluation and Application of Population Pharmacokinetic Models for Identifying Delayed Methotrexate Elimination in Patients With Primary Central Nervous System Lymphoma
Source: Front Pharmacol. 2022 Mar 9;13:817673. doi: 10.3389/fphar.2022.817673 (PMC8959905; doi:10.3389/fphar.2022.817673)

# Electronic Supplementary Material

## Supplementary Figure S1


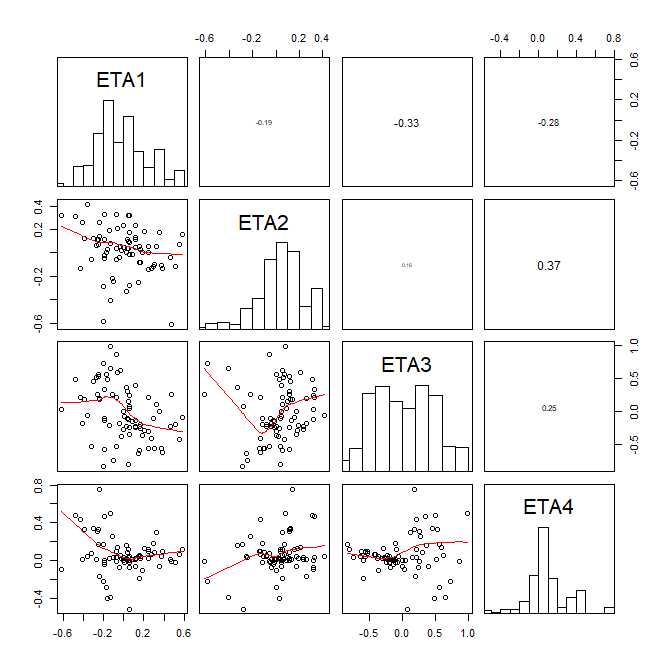


## Supplementary Figure S2


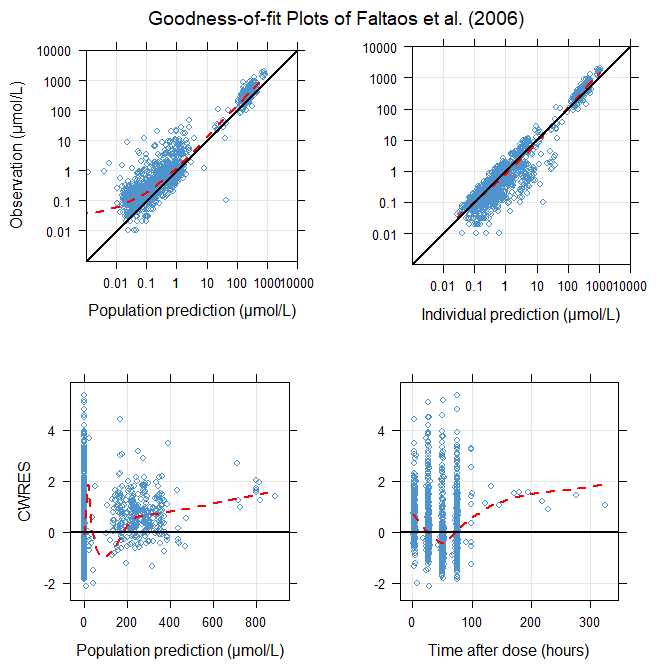


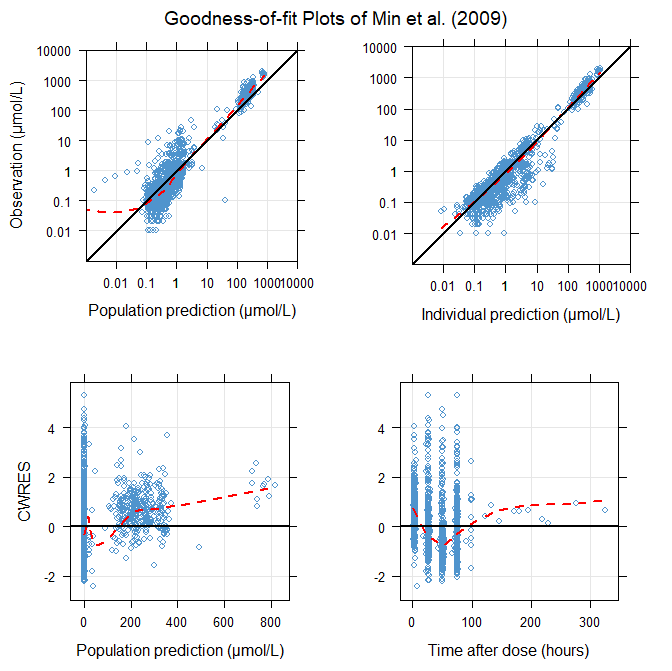


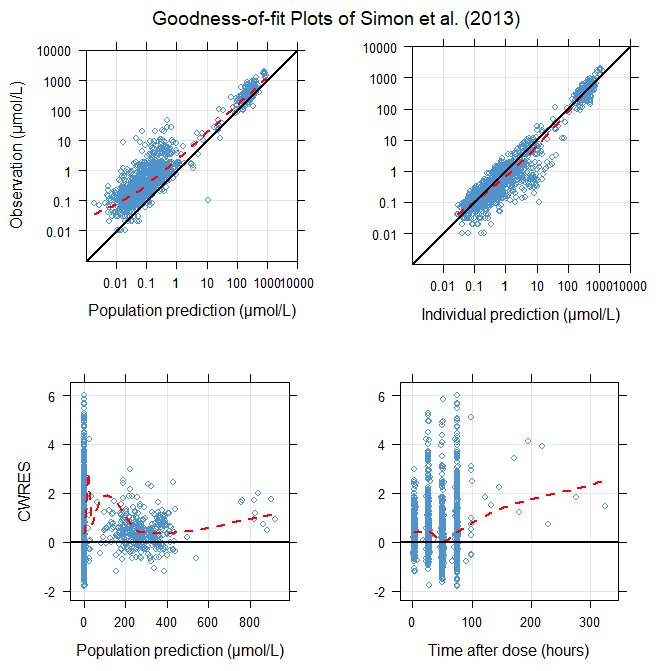


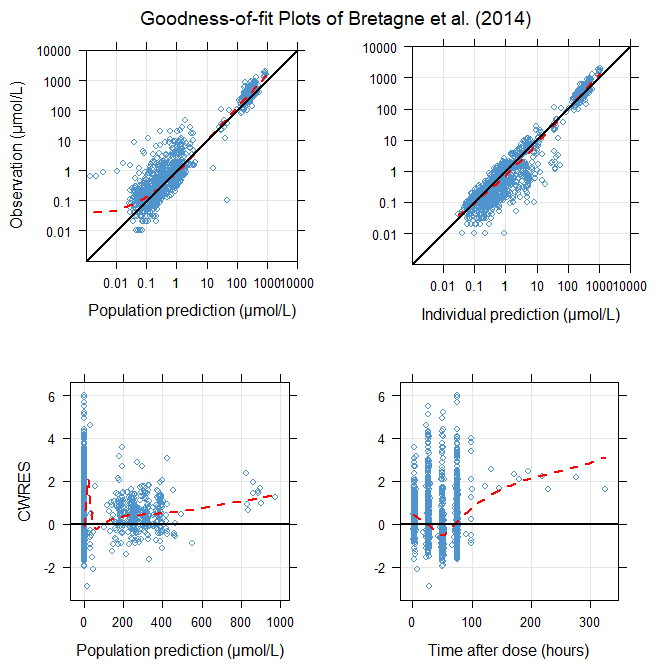


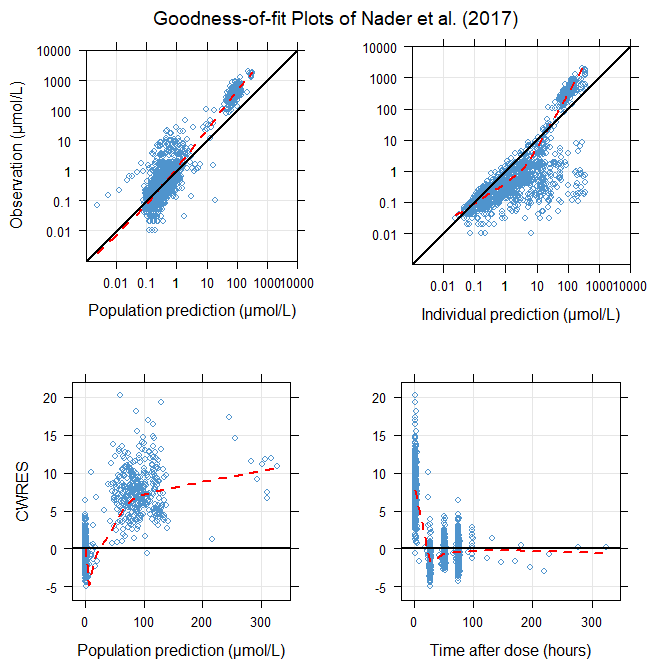


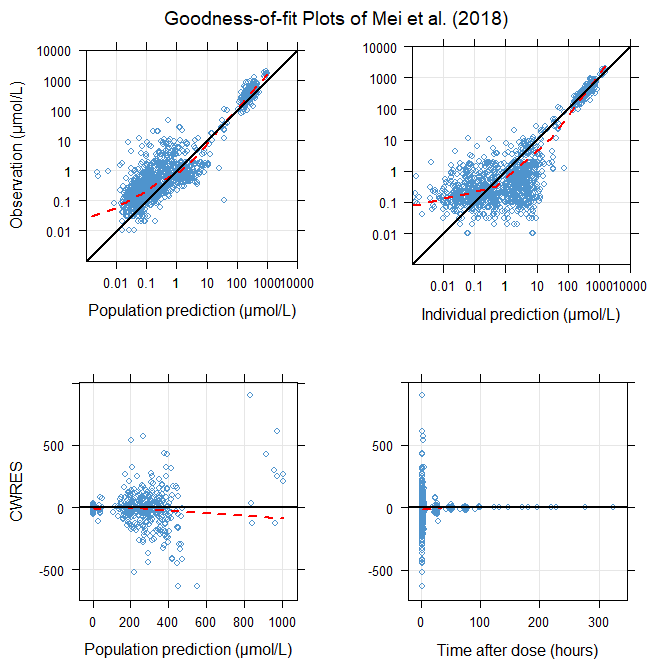


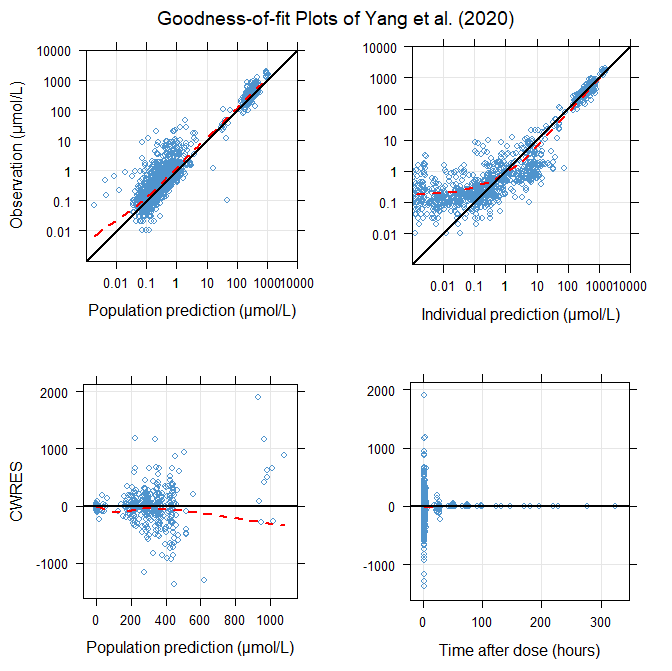


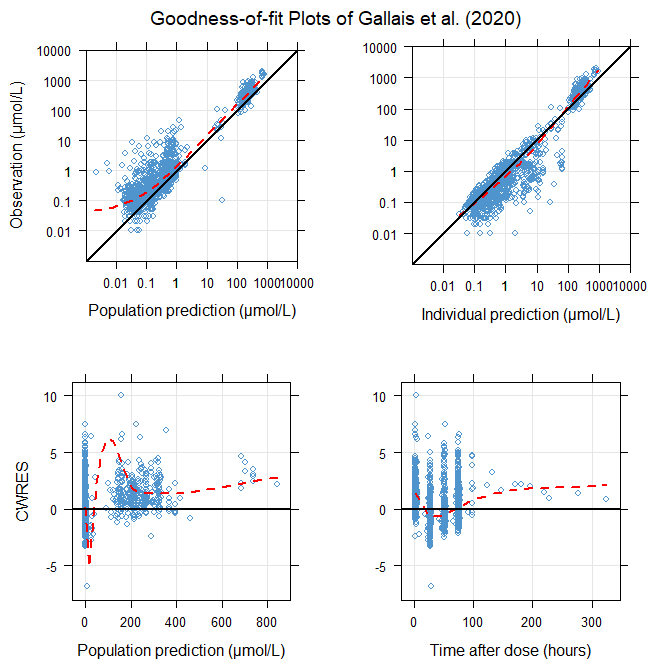


## Supplementary Figure S3


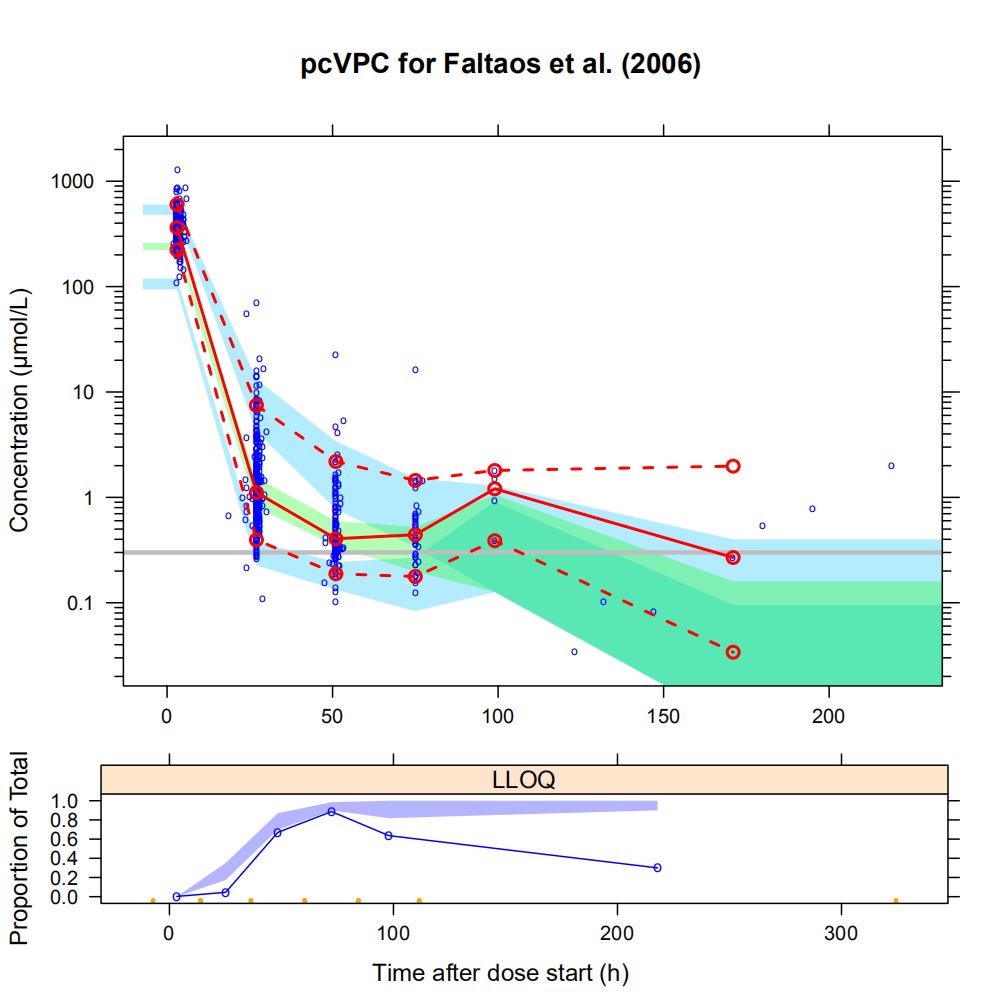


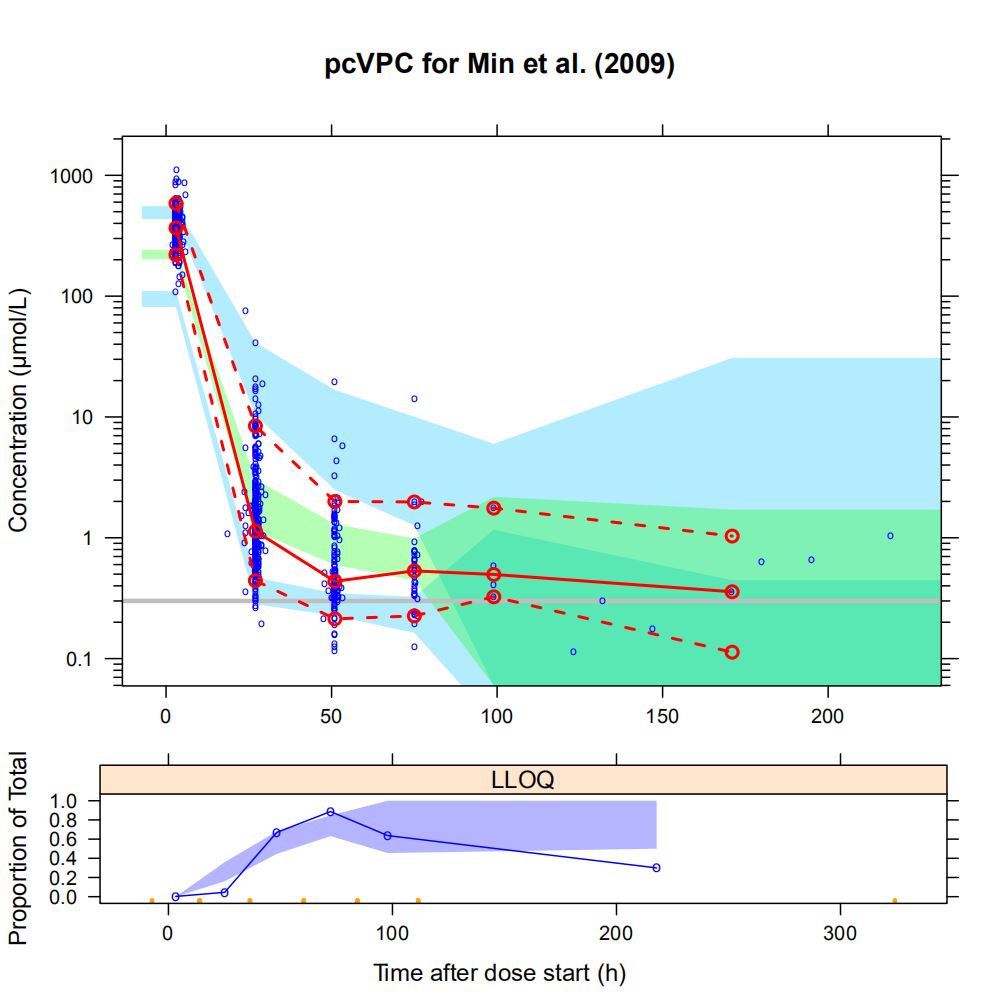


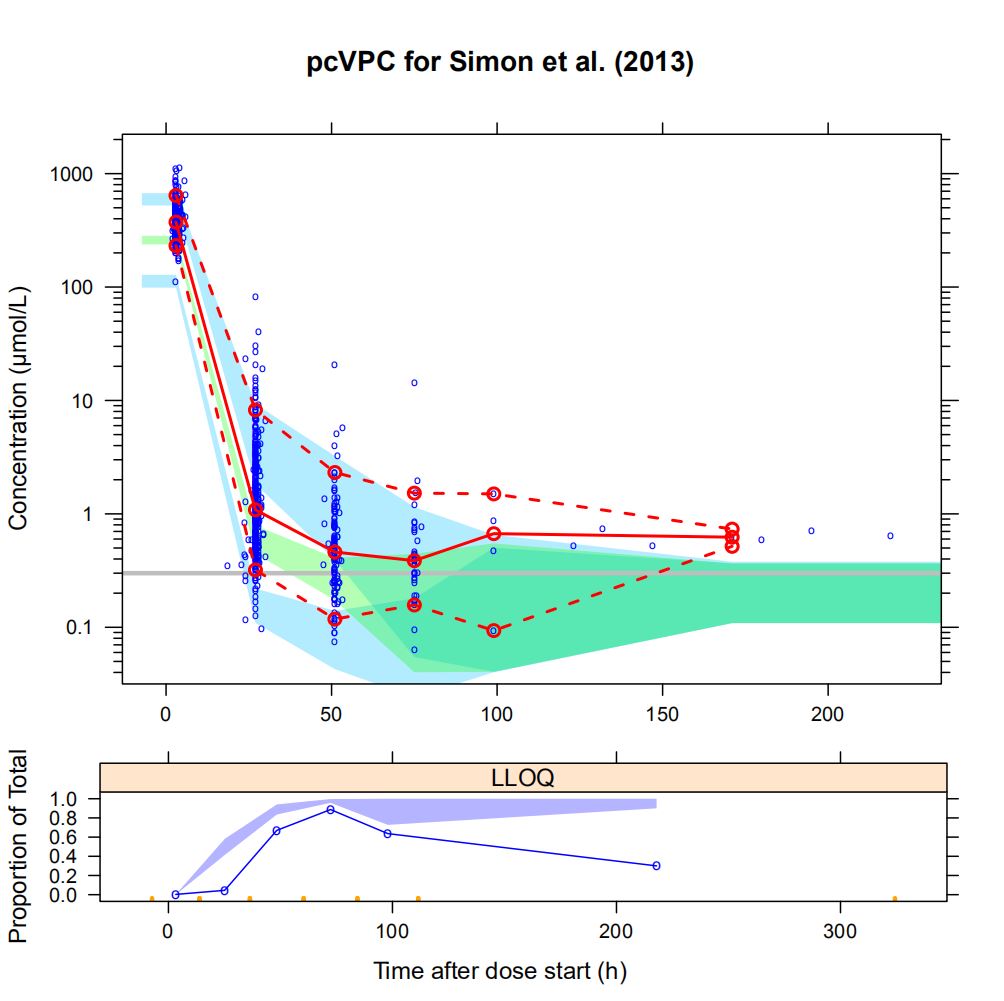


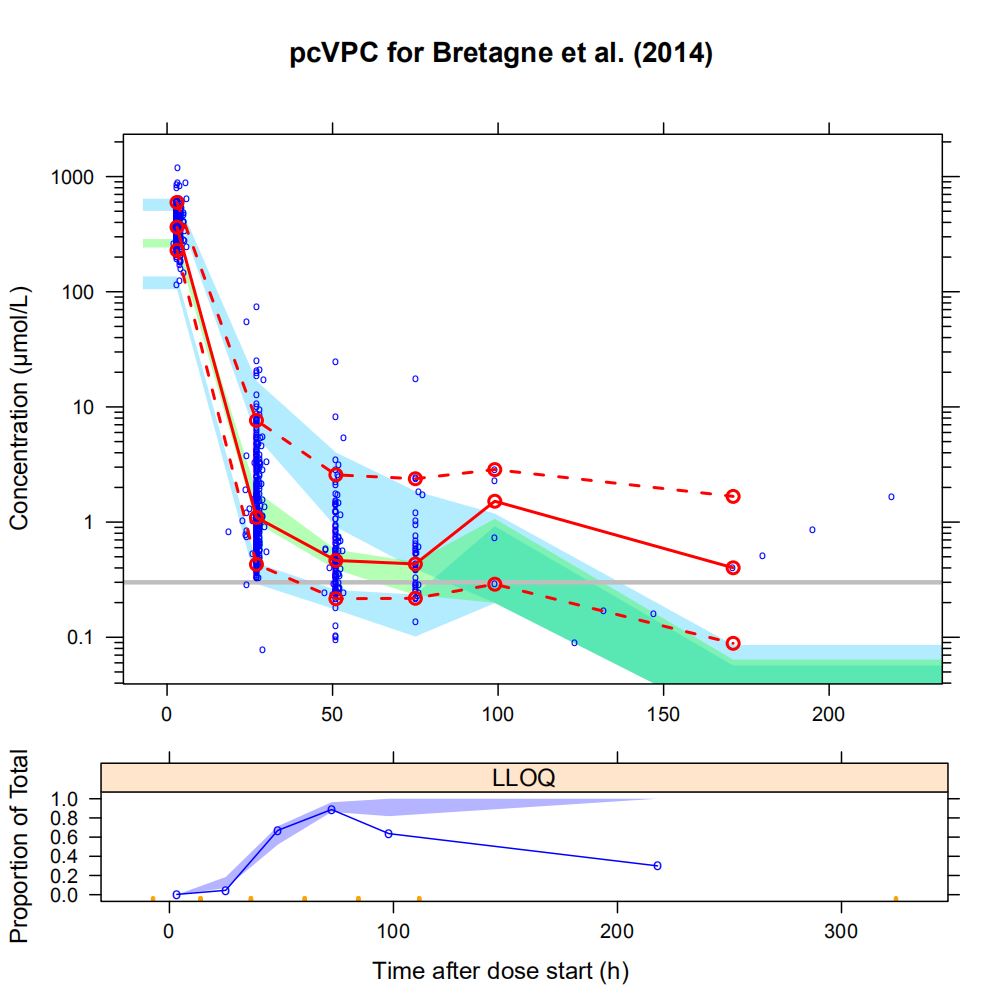


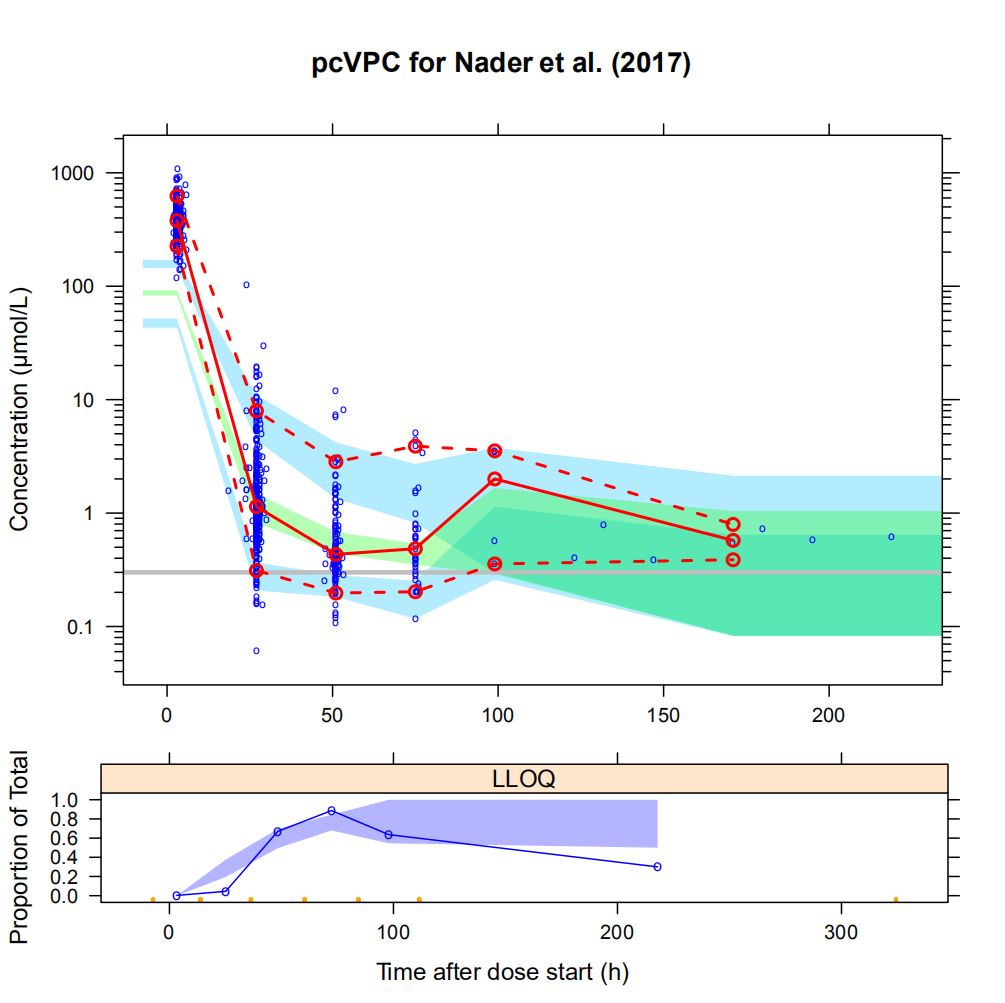


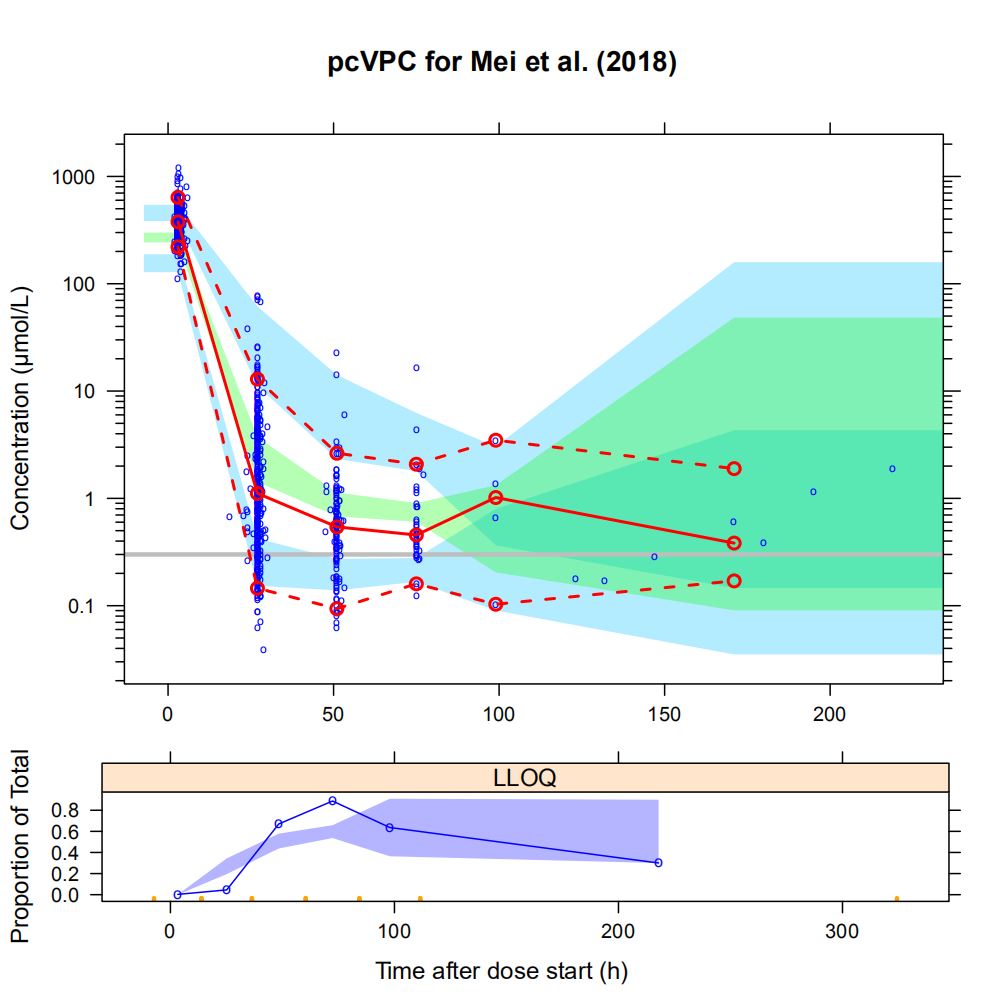


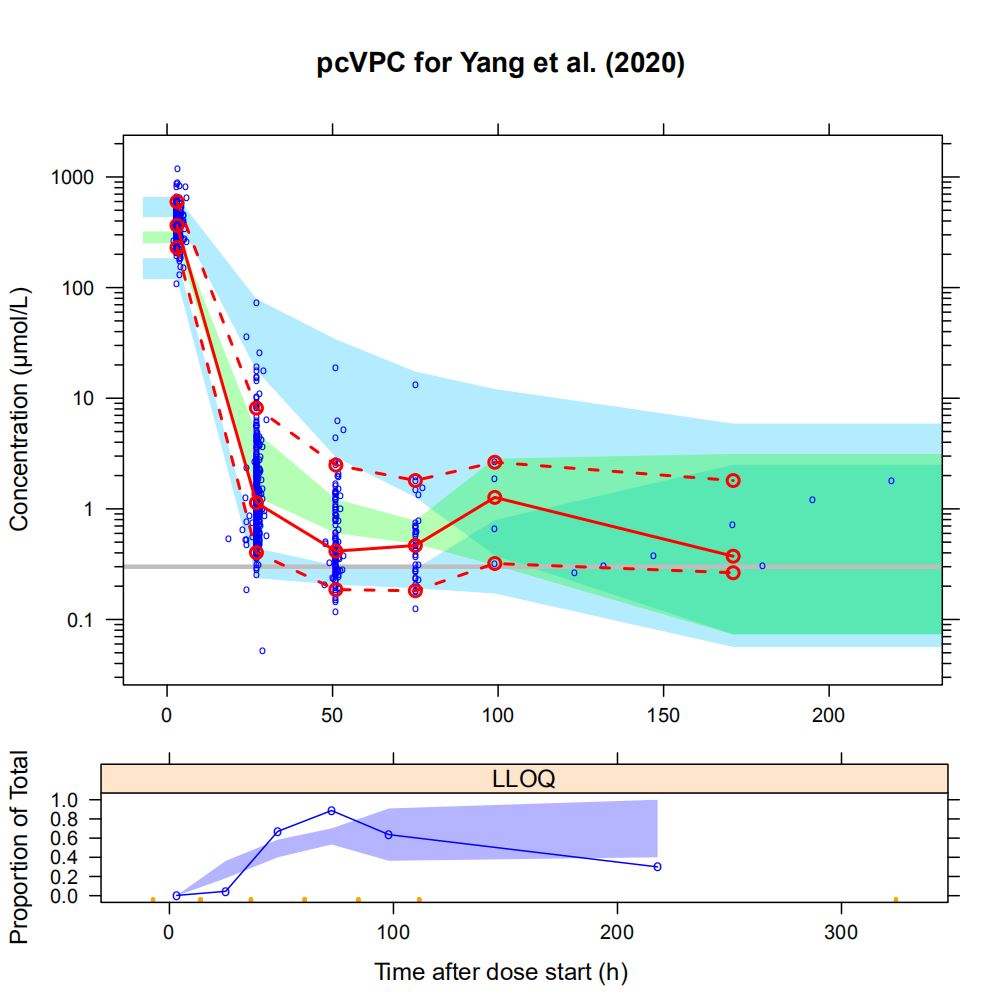


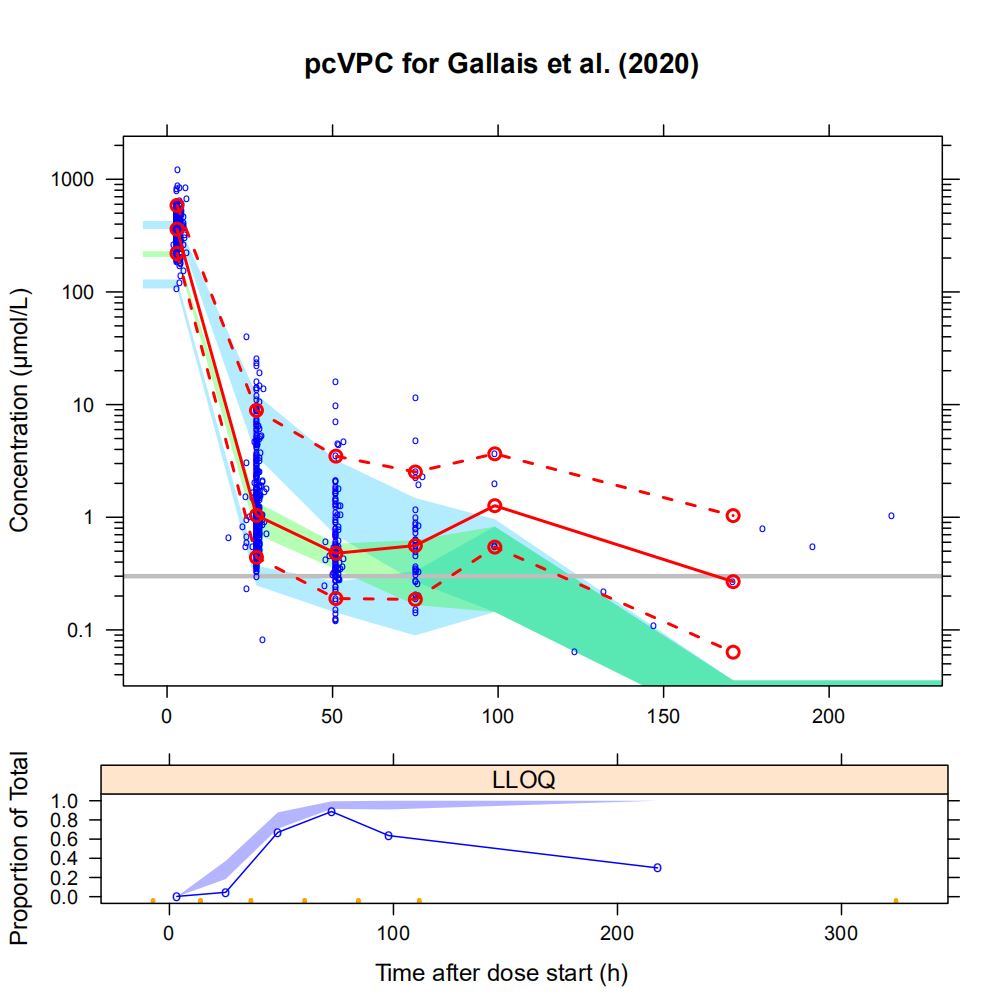

Supplement: Supplementary file 1 [file DataSheet3.docx]
