# Supplementary material for: Evaluation and Application of Population Pharmacokinetic Models for Identifying Delayed Methotrexate Elimination in Patients With Primary Central Nervous System Lymphoma
Source: Front Pharmacol. 2022 Mar 9;13:817673. doi: 10.3389/fphar.2022.817673 (PMC8959905; doi:10.3389/fphar.2022.817673)
Supplement: Supplementary file 2 [file DataSheet2.docx]

# Electronic Supplementary Material

## Supplementary Text S1 Detailed literature search process

Inclusion criteria:

(1) Studied population: adult patients with lymphoid malignancy

(2) Treatment: high-dose methotrexate (HD-MTX)

(3) PK analysis with NONMEM® software package

(4) Language: English

Exclusion criteria:

1. Model parameters were not available for external evaluation and re-estimation
2. Datasets were overlapped or articles were duplicated

Overview of the searching strategy:

241 articles identified by search strategy:

PubMed (n=81); EMbase (n=113); Web of Science (n=47)

Studies remained after database duplicates removed (n=131)

Additionally publications identified from the reference lists of selected papers (n=0)

Studies excluded by reading title and/or abstract (n=120)

Full-text articles retrieved for more detailed judgement (n=11)

Studies excluded due to following reasons^*^:

1. Not analysis with NONMEM® (n = 2)
2. Model parameters not available (n = 1)

Studies involved for external evaluation (n=8)^†^

Searching terms used were:

“ ((population pharmacokinetic*) OR non-linear mixed effect) AND methotrexate”, with * as a wildcard.

^*^Studies excluded (n = 4) due to following reasons:

1. Not analysis with NONMEM® (n=2)

- The study by Pai *et al.* [[1](#_ENREF_1)] was modeling with Monolix2019R2
- The study by Woillard *et al.* [[2](#_ENREF_2)] was modeling with Pmetrics

1. Model parameters not available (n=1)

- The study by Blasco *et al*. [[3](#_ENREF_3)] missed the estimated value of Q/*F*

†In 8 involved studies,

- The genetic information of ABCC2 was not available in external evaluation of Simon *et al.* [[4](#_ENREF_4)]
- DP3, the change of basal value of urinary coproporphyrin I / coproporphyrin I + III ratio at the time of hospital discharge refer to the MTX pre-administration was not available in external evaluation of Bretagne *et al.* [[5](#_ENREF_5)]

## Supplementary Table S1 Drug interaction score of concomitant medications

| Concomitant medications | Drug interaction score |
| --- | --- |
| Omeprazole | 2 |
| Esomeprazole | 2 |
| Lanzoprazole | 2 |
| Pantoprazole | 2 |
| Dexamethasone | 1 |

## Supplementary Table S2 Number of samples and number of samples with concentration below the limit of quantification within each time interval after dose

| Time interval (h) | Mean sampling Time (h) | Number of samples collected | Number of samples below LOQ, n (%) |
| --- | --- | --- | --- |
| 0-36 | 15.4 | 739 | 18 (2.4) |
| 36-60 | 51.2 | 370 | 248 (67.0) |
| 60-84 | 75.1 | 327 | 291 (89.0) |
| 84-108 | 98.3 | 11 | 7 (63.6) |
| >108 | 199.5 | 10 | 3 (30.0) |

LOQ, limit of quantification

## Supplementary Table S3 Summary of covariates screening in our study and 8 investigated published studies

| **Study**  **(year)** | **Covariates screened in data analysis** | **Covariates identified as influencing MTX PK** |
| --- | --- | --- |
| Our study | AGE, GEND, WT, HT, BSA, SCR, CrCL, AST, ALT, ALB, HCT and co-administered drugs | CrCL, ALB, AGE on CL/*F* |
| Faltaos *et al.*  (2006) [[6](#_ENREF_6)] | AGE, GEND, WT, HT, BSA, SCR, CrCL, AST, ALT, GGT, TBIL, ALK, LDH, and proteinemia | AGE, SCR on CL/*F* |
| Min *et al.*  (2009) [[7](#_ENREF_7)] | AGE, GEND, WT, HT, SCR, CrCL, TP, ALT, AST, ALB, TBIL, ALK, LDH and co-administered drugs | SCR on CL/*F*  WT on V_c_/*F* |
| Simon *et al.*  (2013) [[4](#_ENREF_4)] | AGE, GEND, WT, HT, BSA, SCR, CrCL, AST, ALT, GGT, TBIL, ALK, proteinemia and ABCC2 genotyping | CrCL, ABCC2 on CL/*F*  ABCC2 on V_c_/*F* |
| Bretagne *et al.*  (2014) [[5](#_ENREF_5)] | AGE, GEND, WT, HT, BSA, SCR, CrCL, SUR, TP, ALT, AST, ALB, PAL, GGT, LDH, TBIL and co-administered drugs | CrCL, DP3, SCO2 on CL/*F* |
| Nader *et al.*  (2017) [[8](#_ENREF_8)] | AGE, GEND, WT, SCR, BUN, HCT, HB, ALT, AST, ALP, TBIL, and cancer type | HCT on CL/*F*  WT on V_c_/*F* |
| Mei *et al.*  (2018) [[9](#_ENREF_9)] | AGE, GEND, HT, WT, BSA, SCR, CrCL BUN, HCT, HB, TP, GLO, ALT, AST, ALB, and co-administered drugs | SCR, BSA on CL/*F*  AGE on V_c_/*F* |
| Yang *et al.*  (2020) [[10](#_ENREF_10)] | AGE, GEND, HT, WT, BSA, disease stage, SCR, CrCL, BUN, HCT, ALT, AST, ALB and SNPs of FPGS, GGH, SLCO1B1, ABCB1, and MTHFR. | CrCL on CL/*F*  BSA on Q/*F* |
| Gallais *et al.*  (2020) [[11](#_ENREF_11)] | AGE, GEND, HT, WT, BSA, BMI, LBM, IWT, eGFR, SCR, TP, ALT, AST, ALB, GGT, TBIL | AGE on CL/*F*  WT on V_p_/*F* |

ALB, albumin; ALK, alkaline phosphatase; ALT, alanine aminotransferase; AST, aspartate aminotransferase; BMI, body mass index; BSA, body surface area; BUN, blood urea nitrogen; CrCL, creatinine clearance; DP3, the change of basal value of urinary coproporphyrin I/coproporphyrin I + III ratio at the time of hospital discharge refer to the MTX pre-administration; eGFR, estimated GFR according to CKD-EPI formula corrected by individual BSA; FPGS, folylpolyglutamate synthetase; GEND, gender; GGH, gamma-glutamyl hydrolase; GGT, gamma-glutamyl transferase; GLO, globulin; HB, hemoglobin; HCT, heamatocrit; HT, height; IWT, ideal body weight; LBM, lean body mass; LDH, lactate dehydrogenase; MTHFR, methylene tetrahydrofolate reductase; SCO2, co-administered with at least one drug of score 2; SCR, serum creatinine; SUR, serum urea; TBIL, total bilirubin; TP, total protein; WT, body weight

## Supplementary Table S4 Comparison of demographic characteristics of our dataset and 8 investigated published studies

| **Study**  **(year)** | **Age**  **(years)** | **Weight**  **(kg)** | **BSA**  **(m^2^)** | **CrCL**  **(ml min^-1^)** | **ALB**  **(g L^-1^)** |
| --- | --- | --- | --- | --- | --- |
| Our data set | 54.6 ± 9.2/  56 (28-76) | 67.8 ± 10.7/  69.0 (41.0-94.0) | 1.6 ± 0.3/  1.61 (0.85-2.32) | 104.2 ± 34.2/  98 (15.1-326.5) | 38.7 ± 4.3/  39.0 (24.0-50.0) |
| Faltaos *et al.*  (2006) [[6](#_ENREF_6)] | NA/  62 (39-72)^a^ | NA/67  (55-76)^a^ | NA/1.8  (1.6-1.9)^a^ | NA/106  (54-149)^a^ | NA/35  (35-37)^a^ |
| Min *et al.*  (2009) [[7](#_ENREF_7)] | 40 ± 18.7/  37 (3-80) | 66.0 ± 14.1/  64.8 (17.0-115.0) | NA | 107.4 ± 37.5/  104.4 (25.6-200.4) | 38.2 ± 5.2/  39.0 (23.1-49.7) |
| Simon *et al.*  (2013) [[4](#_ENREF_4)] | NA/  62 (20-81) | NA/  67 (40-98) | NA/  1.81 (1.4-2.1) | NA/  5.1 (4-13) | NA/  35 (28-48) |
| Bretagne *et al.*  (2014) [[5](#_ENREF_5)] | NA/  60.6 (18.8-84.6) | NA/  75 (43-110) | NA/  1.86 (1.34-2.29) | NA/  91.6 (36.1-257.0) | NA/  39 (21-46) |
| Nader *et al.*  (2017) [[8](#_ENREF_8)] | 35 ± 12/  (14-66) | 69 ± 15/  (50-109) | NA | NA | NA |
| Mei *et al.*  (2018) [[9](#_ENREF_9)] | 52.1 ± 18.5/  (8.5-83.6) | 63.7 ± 12.0/  (28.5-94) | 1.73 ± 0.24/  (1.10-2.33) | 99.47 ± 35.88/  (27.05-294.81) | 37.81 ± 3.63/  (22.7-51.6) |
| Yang *et al.*  (2020) [[10](#_ENREF_10)] | NA / 48  (18-73) | NA / 61  (44-90) | NA / 1.65  (1.28-2.06) | NA / 115.1  (60-153.4) | NA / 37.9  (21.5-50) |
| Gallais *et al.*  (2020) [[11](#_ENREF_11)] | 49.8 ± 17.1/  (17.8-83.6) | 70.2 ± 15.5/  (41-136) | 1.8 ± 0.2 /  (1.4-2.5) | NA | 31.7 ± 5.2 /  (14-59) |

ALB, Albumin; BSA, body surface area; CrCL, creatinine clearance; NA, not available

Data is expressed as Mean ± SD / Median (Range), if applicable

^a^ Data is expressed as 25^th^ - 75^th^ percentile

## Supplementary Table S5 The results of covariates screen procedure

| Model No. | Model description | OFV | ΔOFV | *p* value | Results |
| --- | --- | --- | --- | --- | --- |
| *Forward inclusion* | | | | | |
| 1 | Base model | 4306.6 | / | / |  |
| 2 | Add CrCL on CL in model 1 | 4209.0 | -97.6 | *p* < 0.001 | YES |
| 3 | Add ALB on CL in model 2 | 4149.1 | -59.9 | *p* < 0.001 | YES |
| 4 | Add HCT on CL in model 2 | 4194.2 | -14.8 | *p* < 0.001 | NO |
| 5 | Add AGE on CL in model 3 | 4135.8 | -13.3 | *p* < 0.001 | YES |
| 6 | Add WT on PK parameters in model 5 allometrically | 4132.0 | -3.8 | *p* < 0.001 | NO |
| 7 | Add BSA on PK parameters in model 5 allometrically | 4132.1 | -3.73 | *p* < 0.001 | NO |
| 8 | Add BMI on PK parameters in model 5 allometrically | 4144.4 | 8.58 | *p* < 0.001 | NO |
| 9 | Add DIS ≥ 2 on CL in model 5 using a shift model | 4132.2 | -3.6 | *p* < 0.001 | NO |
| *Backward elimination* | | | | | |
| 10 | Remove CrCL on CL from model 5 | 4261.1 | 125.3 | *p* < 0.001 | NO |
| 11 | Remove ALB on CL from model 5 | 4160.9 | 25.1 | *p* < 0.001 | NO |
| 12 | Remove AGE on CL from model 5 | 4149.1 | 13.4 | *p* < 0.001 | NO |

ALB, Albumin; BMI, body mass index; BSA, body surface area; CL, apparent clearance; CrCL, creatinine clearance; DIS, drug interaction score; HCT, hematocrit; OFV, objective function value; WT, body weight

## Supplementary Table S6 Characteristics of involved covariates levels in simulation

|  | 2.5^th^ Percentile | Median | 97.5^th^ Percentile |
| --- | --- | --- | --- |
| Creatinine Clearance (ml min^-1^) | 46.3 | 98 | 188 |
| Albumin (g L^-1^) | 29 | 39 | 47 |

Patients with median covariates were considered as standard

## Supplementary Table S7 The proportion of patients with delayed elimination in different scenarios

| Simulation  scheme | AGE  (year) | ALB  (g L^-1^) | CrCL  (ml min^-1^) | The proportion of patients had MTX concentrations below 0.2 μmol/L at 72h (%) |
| --- | --- | --- | --- | --- |
| 01a | < 60 | 29 | 46.3 | 29.6 |
| 01b |  | 29 | 98 | 66.6 |
| 01c |  | 29 | 188 | 88.7 |
| 02a | ≥ 60 | 29 | 46.3 | 18.6 |
| 02b |  | 29 | 98 | 54.8 |
| 02c |  | 29 | 188 | 81.3 |
| 03a | < 60 | 39 | 46.3 | 38.2 |
| 03b |  | 39 | 98 | 74.5 |
| 03c |  | 39 | 188 | 92.3 |
| 04a | ≥ 60 | 39 | 46.3 | 27.6 |
| 04b |  | 39 | 98 | 64.7 |
| 04c |  | 39 | 188 | 87.4 |
| 05a | < 60 | 47 | 46.3 | 44.8 |
| 05b |  | 47 | 98 | 78.7 |
| 05c |  | 47 | 188 | 94.3 |
| 06a | ≥ 60 | 47 | 46.3 | 33.3 |
| 06b |  | 47 | 98 | 70.9 |
| 06c |  | 47 | 188 | 90.9 |

ALB, Albumin; CrCL, creatinine clearance

**Reference**

1. Pai MP, Debacker KC, Derstine B, Sullivan J, Su GL, Wang SC. Comparison of Body Size, Morphomics, and Kidney Function as Covariates of High-Dose Methotrexate Clearance in Obese Adults with Primary Central Nervous System Lymphoma. Pharmacotherapy 2020; 40: 308-19.

2. Woillard JB, Debord J, Benz-de-Bretagne I, Saint-Marcoux F, Turlure P, Girault S, Abraham J, Choquet S, Marquet P, Barin-Le Guellec C. A Time-Dependent Model Describes Methotrexate Elimination and Supports Dynamic Modification of MRP2/ABCC2 Activity. Therapeutic drug monitoring 2017; 39: 145-56.

3. Blasco H, Senecal D, Le Gouge A, Pinard E, Benz-de Bretagne I, Colombat P, Hulot JS, Chatelut E, Le Guellec C. Influence of methotrexate exposure on outcome in patients treated with MBVP chemotherapy for primary central nervous system lymphoma. British journal of clinical pharmacology 2010; 70: 367-75.

4. Simon N, Marsot A, Villard E, Choquet S, Khe HX, Zahr N, Lechat P, Leblond V, Hulot JS. Impact of ABCC2 polymorphisms on high-dose methotrexate pharmacokinetics in patients with lymphoid malignancy. The pharmacogenomics journal 2013; 13: 507-13.

5. Benz-de Bretagne I, Zahr N, Le Gouge A, Hulot JS, Houillier C, Hoang-Xuan K, Gyan E, Lissandre S, Choquet S, Le Guellec C. Urinary coproporphyrin I/(I + III) ratio as a surrogate for MRP2 or other transporter activities involved in methotrexate clearance. British journal of clinical pharmacology 2014; 78: 329-42.

6. Faltaos DW, Hulot JS, Urien S, Morel V, Kaloshi G, Fernandez C, Xuan k H, Leblond V, Lechat P. Population pharmacokinetic study of methotrexate in patients with lymphoid malignancy. Cancer chemotherapy and pharmacology 2006; 58: 626-33.

7. Min Y, Qiang F, Peng L, Zhu Z. High dose methotrexate population pharmacokinetics and Bayesian estimation in patients with lymphoid malignancy. Biopharmaceutics & drug disposition 2009; 30: 437-47.

8. Nader A, Zahran N, Alshammaa A, Altaweel H, Kassem N, Wilby KJ. Population Pharmacokinetics of Intravenous Methotrexate in Patients with Hematological Malignancies: Utilization of Routine Clinical Monitoring Parameters. Eur J Drug Metab Pharmacokinet 2017; 42: 221-28.

9. Mei S, Li X, Jiang X, Yu K, Lin S, Zhao Z. Population Pharmacokinetics of High-Dose Methotrexate in Patients With Primary Central Nervous System Lymphoma. Journal of pharmaceutical sciences 2018; 107: 1454-60.

10. Yang L, Wu H, de Winter BCM, Sheng CC, Qiu HQ, Cheng Y, Chen J, Zhao QL, Huang J, Jiao Z, Xie RX. Pharmacokinetics and pharmacogenetics of high-dose methotrexate in Chinese adult patients with non-Hodgkin lymphoma: a population analysis. Cancer chemotherapy and pharmacology 2020; 85: 881-97.

11. Gallais F, Oberic L, Faguer S, Tavitian S, Lafont T, Marsili S, Brice A, Chatelut E, Puisset F. Body Surface Area Dosing of High-Dose Methotrexate Should be Reconsidered, Particularly in Overweight, Adult Patients. Therapeutic drug monitoring 2020
