# Supplementary material for: Evaluation and Application of Population Pharmacokinetic Models for Identifying Delayed Methotrexate Elimination in Patients With Primary Central Nervous System Lymphoma
Source: Front Pharmacol. 2022 Mar 9;13:817673. doi: 10.3389/fphar.2022.817673 (PMC8959905; doi:10.3389/fphar.2022.817673)
Supplement: Supplementary file 3 [file DataSheet1.docx]

# Supporting information

Additional Supporting Information may be found in the online version of this article at the publisher’s web-site:

***Supplementary Text S1*** Detailed literature search process

***Table S1*** Drug interaction score of concomitant medications

***Table S2*** Number of samples and number of samples with concentration below the limit of quantification within each time interval after dose

***Table S3*** Summary of covariates screening in our study and 8 investigated published studies

***Table S4*** Comparison of demographic characteristics of our dataset and 8 investigated published studies

***Table S5*** The results of covariates screen procedure

***Table S6*** Characteristics of involved covariates levels in simulation

***Table S7*** The proportion of patients with delayed elimination in different scenarios

***Figure S1*** Correlation charts for BSA of each PK parameters. The *red line* represents the LOESS smoothing, and the number in upper right represent the correlation coefficient between covariates. ETA1, ETA2, ETA3, ETA4 present the between-subject variability of apparent clearance, apparent central volume of distribution, inter-compartmental clearance and apparent peripheral volume of distribution, respectively.

***Figure S2*** Diagnostic goodness-of-fit plots for the 8 investigated published models

***Figure S3*** Visual predictive checks (VPCs) for the 8 investigated published models, based on 2000 simulations
